# Integrated single-cell transcriptomic and epigenetic study of cell state transition and lineage commitment in embryonic mouse cerebellum

**DOI:** 10.1126/sciadv.abl9156

**Published:** 2022-04-01

**Authors:** Nagham Khouri-Farah, Qiuxia Guo, Kerry Morgan, Jihye Shin, James Y. H. Li

**Affiliations:** 1 Department of Genetics and Genome Sciences, University of Connecticut School of Medicine, 263 Farmington Avenue, Farmington, CT 06030-6403, USA.; 2 Institute for Systems Genomics, University of Connecticut, 400 Farmington Avenue, Farmington, CT 06030-6403, USA.

## Abstract

Recent studies using single-cell RNA-sequencing have revealed cellular heterogeneity in the developing mammalian cerebellum, yet the regulatory logic underlying this cellular diversity remains to be elucidated. Using integrated single-cell RNA and ATAC analyses, we resolved developmental trajectories of cerebellar progenitors and identified putative trans- and cis-elements that control cell state transition. We reverse engineered gene regulatory networks (GRNs) of each cerebellar cell type. Through in silico simulations and in vivo experiments, we validated the efficacy of GRN analyses and uncovered the molecular control of a posterior transitory zone (PTZ), a distinct progenitor zone residing immediately anterior to the morphologically defined rhombic lip (RL). We showed that perturbing cell fate specification in the PTZ and RL causes posterior cerebellar vermis hypoplasia, the most common cerebellar birth defect in humans. Our study provides a foundation for comprehensive studies of developmental programs of the mammalian cerebellum.

## Teaser

Single-cell genomics and machine learning reveal a potential mechanism leading to the most common cerebellar birth defect.

## INTRODUCTION

The cerebellum, which contains 80% of the total neurons of the human brain, is important in cognitive processing and sensory discrimination, in addition to its well-known function in motor coordination. There is a resurgence of interest in the development of the cerebellum as it is recognized as a locus for numerous developmental brain disorders, such as ataxia, autism, schizophrenia, and attention-deficit/hyperactivity disorder (*1*).

The embryonic cerebellum contains two spatially distinct germinal zones: the ventricular zone (VZ) and the upper rhombic lip (RL), which give rise to inhibitory and excitatory neurons, respectively (*2*). From these germinal zones, different types of GABAergic and glutamatergic neurons are generated in temporally restricted phases (*2*). In particular, the RL produces cerebellar glutamatergic nuclear neurons between embryonic day (E) 9.5 and E12.5, granule cells (GCs) after E12.5, and unipolar brush cells at E16.5 (*2*). Genetic studies have demonstrated that transcription factor (TF) Ptf1a controls the GABAergic fate of VZ progenitors, whereas Atoh1 controls the glutamatergic fate of RL cells (*2*). Despite these advances, the gene regulatory programs that regulate the diversification of GABAergic and glutamatergic neurons remain to be defined.

It was suggested that RL-derived *Atoh1* cells are induced de novo throughout embryogenesis (*3*). However, the molecular mechanisms responsible for the remarkable cellular diversity of the *Atoh1* lineage are poorly understood. Furthermore, the source of progenitors that are recruited into the *Atoh1* lineage, particularly those forming cerebellar GCs—the largest population of neurons in the mammalian brain—is still an enigma. We have recently proposed that the posterior end of the cerebellar VZ, named as “posterior transitory zone (PTZ),” contains bipotent progenitor cells for the *Atoh1* lineage and choroid plexus epithelium (*4*). However, the regulation and function of the PTZ have not been examined. A recent report showed that the RL in the developing human cerebellum has distinctive cytoarchitectural features from other vertebrates, including the rhesus macaque (*5*). Unusual longevity of the human RL is attributed to the expansion of the posterior cerebellar vermis, a region that is associated with human cognition and predominantly affected in cerebellar birth defects such as Dandy-Walker malformation (DWM) and cerebellar vermis hypoplasia (*5*). Therefore, studying the molecular and cellular mechanisms that sustain the *Atoh1* lineage will shed light on the evolution and congenital defects of the human cerebellum.

C. Waddington introduced the concept of epigenomic landscape to explain the emergence of distinct cell fates during development (*6*). In particular, the chromatin state defines the functional architecture of the genome by modulating the accessibility of cis-regulatory elements (CREs), which are regions of DNA bound by TFs to regulate the transcription of target genes. Together, the CREs and TFs constitute the regulatory logic for cell state transition and lineage commitment. The adaptation of assay for transposase accessible chromatin (ATAC) allows measuring of chromatin accessibility, a proxy for CRE activity, at single-cell resolution (*7*). Powerful algorithms have been developed to harness time-series single-cell data to identify the molecular trajectories that describe cell fate specification in vertebrate embryogenesis (*8*–*10*). Although several studies using single-cell technology have examined the heterogeneous cell populations in the mouse (*4*, *11*–*15*) and human cerebellum (*16*), systematic integrated RNA and ATAC analysis by sampling developing cerebellar cells is still lacking.

In the current study, we applied single-cell RNA-sequencing (scRNA-seq) to resolve the developmental trajectories and the underlying transcriptional changes in the embryonic mouse cerebellum. We profiled the accessible chromatin of the mouse cerebellum at E12.5, E13.5, and E14.5 using single-nucleus ATAC sequencing (snATAC-seq). By integrating single-cell resolved RNA and ATAC modalities, we identified CREs that undergo temporal, cell type–specific changes in chromatin accessibility and linked them to transcriptional targets. On the basis of information on the trans- and cis-elements and their linked targets, we reverse engineered gene regulatory networks (GRNs) of individual cerebellar cell types and applied the GRNs to simulate loss of function and gain of function (GOF) of transcription regulators. Last, we performed genetic and fate-mapping studies to validate our in silico analyses. We refined the molecular features of the PTZ, a VZ domain immediately anterior to the classically defined RL. We showed that the balance of cell fate specification in the PTZ and RL is crucial for the expansion of the posterior cerebellar vermis in mice. Several genes that have been implicated in DWM, the most common congenital human cerebellar defect with cerebellar vermis hypoplasia, are enriched in the PTZ, suggesting that abnormal development of PTZ cells underlies the pathogenesis of DWM.

## RESULTS

### Reconstruction of cerebellar development with time-series scRNA-seq

**Fig. 1.**
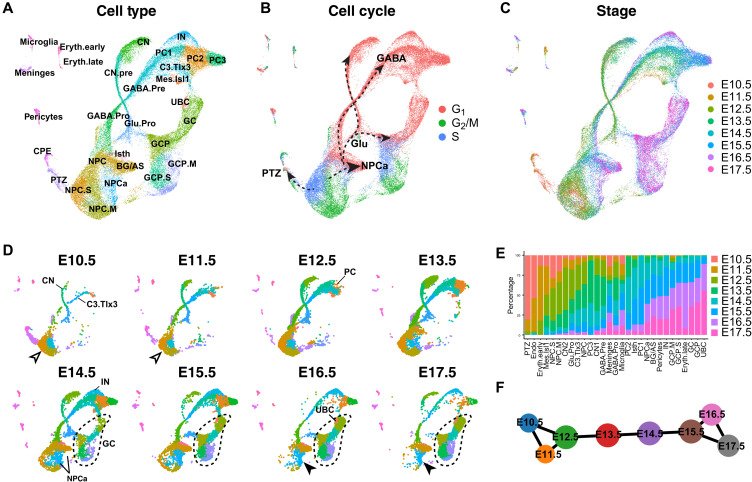
Reconstruction of the development of the embryonic mouse cerebellum by time-series scRNA-seq. (**A** to **D**) UMAP representation of merged scRNA-seq of mouse cerebella from E10.5 to E17.5. Dots indicate individual cells; colors show cell clusters (A and D), cell cycle phases (B), and embryonic stages (C); the dashed-line arrows in (B) denote the main branches of developmental trajectories arising from NPCs; and the empty and black arrowheads in (D) indicate the early and late NPCs, respectively. The initial appearance of cerebellar nuclear neurons (CN), parabrachial nuclear neurons (C3.Tlx3), PCs, IN, cerebellar GCs, and unipolar brush cells (UBC) are indicated in (D). Dashed lines in (D) circle GCs. (**E**) Bar plots showing cell composition across stages. (**F**) Partition-based graph abstraction (PAGA) showing the relationship of cells from different stages. BG/AS, Bergmann glia and astrocytes; CPE, choroid plexus epithelium; Eryth.early and Eryth.late, early and late erythrocytes; GABA.Pro, GABAergic neuron progenitors; GABA.Pre, GABAergic neuron precursors; Glu.Pro, glutamatergic neuron progenitors; Isth, isthmus; NPC.M and NPC.S, NPCs in the M and S phase.

To investigate developmental trajectories of cerebellar neural progenitor cells (NPCs), we reanalyzed a previously published scRNA-seq dataset (*11*), which includes 12 time points from E10.5 to postnatal day (P) 10 mouse cerebella, focusing on the embryonic data (E10.5 to E17.5). On the basis of the known markers, we assigned identities to 29 recovered clusters, which included all major cerebellar cell types (Fig. 1A, fig. S1, and data S1). In Uniform Manifold Approximation and Projection (UMAP) embedding, proliferative cells, including NPCs and granule cell progenitors (GCPs), at either the synthesis (S) or the mitosis (M-G_2_) phase are arranged in circles, resembling the cell cycle, and linked to branches of postmitotic cells (Fig. 1B). Cells from different stages are well integrated, but their relative contributions to each cell type change in agreement with the temporal development of the cell type (Fig. 1, C to E) (*2*). For example, Purkinje cells (PCs) initially appear at E12.5, and GABAergic interneurons (IN) and GCPs first appear at E14.5 and greatly expand afterward (Fig. 1, D and E). The UMAP projection displays four main branches, corresponding to glutamatergic neurons, GABAergic neurons, PTZ, and anterior neural progenitor (NPCa), respectively (Fig. 1B). After E13.5, NPCs shift toward the NPCa, displaying a transition from neural epithelium to radial glia with increasing expression of *Fabp7*, *Lxn*, *Aldh1l1*, and *Slc1a3* (Fig. 1, C and D, and data S1). Along the glutamatergic branch, cells from the early stages form a continuum leading to cerebellar and extra–cerebellar nuclei (CNs), whereas cells after E13.5 contribute to GCs (Fig. 1, C to E), in agreement with a CN-to-GC switch in the production of glutamatergic cerebellar neurons (*3*). Therefore, our scRNA-seq trajectory analyses reconstruct the order of cell differentiation in the embryonic mouse cerebellum.

### Resolution of four developmental trajectories from the cerebellar VZ progenitors

**Fig. 2.**
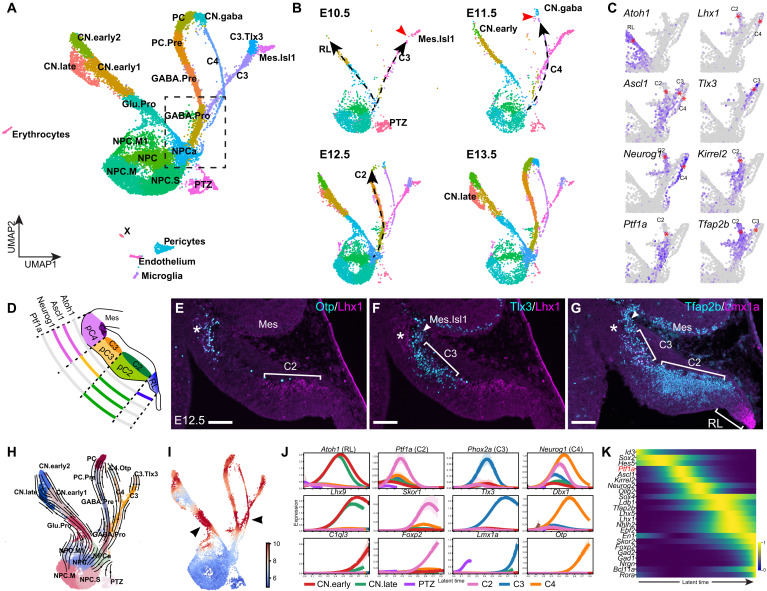
Cell fate choices during early cerebellar development. (**A** and **B**) UMAP projection of cells from the merged E10.5 to E13.5 dataset (A) and the individual stage (B). Dashed line arrows indicate the stepwise appearance of the trajectories; the red arrowheads denote the gap along the trajectory. (**C**) Expression of selected markers in the boxed UMAP area shown in (A). The asterisks denote the branch with the indicated gene. (**D**) Illustration of the regionalization of cerebellar VZ defined by the differential expression of proneural genes. (**E** to **G**) Immunofluorescence on sagittal sections of E12.5 mouse cerebella. Brackets demarcate C2, C3, and the RL; arrowheads indicate Isl1^+^ cells originated from the mesencephalon (Mes); asterisks denote the C4 region, which is positive for Lhx1/5 but negative for Lmx1a, in concordance with the expression pattern shown in (C). Scale bars, 100 μm. (**H** and **I**) scVelo-inferred RNA velocity streamlines (H) and developmental kinetics (I) visualized in a UMAP embedding. Arrowheads indicate regions with accelerated differentiation. (**J**) Expression trends of selected genes across latent time for each developmental trajectory arising from cerebellar NPCs. (**K**) Heatmap showing the expression dynamics of regulators leading to the generation of PCs.

Unbiased hierarchical analysis using partition-based graph abstraction (PAGA), which provides an interpretable graph-like map of the data manifold based on the connectivity of cell groups (*17*), correctly orders cells from E10.5 to E17.5 (fig. S2A). Furthermore, PAGA reveals that cerebellar cells of the early (E10.5 to E12.5) and late (E15.5 to E17.5) embryonic stages are interconnected, whereas cells between E12.5 and E15.5 form a linear connection (Fig. 1F). This tripartite pattern suggests that the cell state transition is dynamic and abrupt between E12.5 and E15.5, but it is much more gradual and asynchronous before and after that. We reasoned that, by taking advantage of the gradual and asynchronous development at the early phase, we could resolve the developmental trajectories from cerebellar NPCs in greater detail. UMAP embedding of E10.5 to E13.5 data reveals four conspicuous trajectories (RL and p2-4), each of which emerges in a stepwise manner (Fig. 2, A and B). Inspecting selected genes revealed that these trajectories represent neurons arising from four cerebellar VZ domains that are demarcated by proneural genes *Atoh1*, *Ascl1*, *Ptf1a*, and *Neurog1*, respectively (Fig. 2, C and D) (*18*, *19*). The glutamatergic and GABAergic lineages are differentially marked by *Atoh1* and *Ascl1*; the latter gives rise to three branches, in which pC2 and pC4 are both marked by *Neurog1*, while pC2 is selectively marked by *Ptf1a* (Fig. 2, C and D). RL and pC2 cells give rise to the cerebellar glutamatergic and GABAergic neurons, respectively (*18*, *19*). pC3 cells are precursors of the parabrachial nuclei (*20*), whereas the fate of pC4 cells is currently unknown. By performing immunofluorescence for the markers that represent each branch, we confirmed the cell types arising from the four cerebellar VZ domains (Fig. 2, E to G).

To study cellular differentiation kinetics, we calculated RNA velocity with scVelo (*21*). Adding RNA velocity vector streams to the UMAP embedding demonstrates the developmental trajectories—the proliferating state of NPCs traverses through the four branches (Fig. 2H). scVelo predicts that the differentiation progression is slow immediately after cell cycle exit and speeds up after cell fate commitment to the individual trajectory (Fig. 2I). Next, we identified cell fates, gene cascades, and driver genes along each developmental trajectory with CellRank (Fig. 2, J and K; fig. S2, B and C; and data S2) (*22*). To validate the analysis, we performed functional enrichment analysis reasoning that genes in a given cascade should have common biological functions. Driver genes of each lineage exhibit distinct enrichments in biological function and pathways (fig. S2, D and E).

To assess the robustness of our results, we examined additional scRNA-seq datasets. First, we repeated the analysis by adding E14.5 cells to the E10.5 to E13.5 dataset. We also reanalyzed another published scRNA-seq data of E10.5, E12.5, and E14.5 mouse cerebella (*14*). Last, we generated our scRNA-seq data of E12.5, E13.5, and E14.5 cerebella (in-house). All these datasets produced almost identical results to the E10.5-to-E13.5 one (fig. S3). Collectively, our scRNA-seq analyses confirm the four subdomains in the cerebellar VZ in mouse embryos between E10.5 and E13.5. We identify the genetic cascades, including putative driver genes, in the four populations of cells arising from the respective cerebellar VZ subdomains.

### Molecular control of cerebellar GABAergic neuron differentiation

**Fig. 3.**
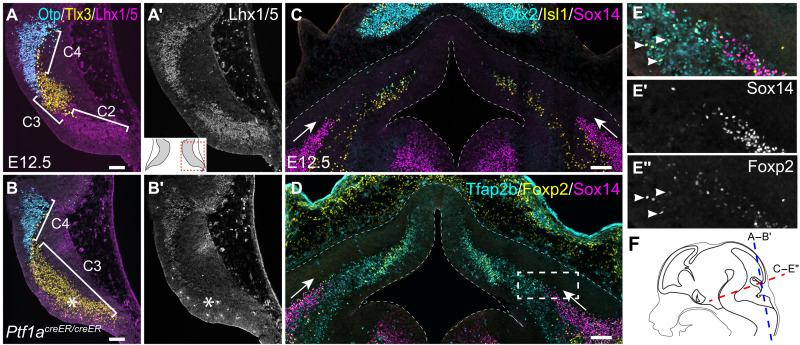
In vivo development of cerebellar GABAergic neurons. (**A** to **B′**) Immunofluorescence on horizontal sections of wild-type and *Ptf1a*-deficient cerebellar anlage at E12.5 (inset illustrating the cerebellar section and the shown area). The asterisks indicate that the loss of Lhx1/5 accompanies an ectopic expression of Tlx3 (*n* = 3). Note that the antibodies detect both Lhx1 and Lhx5. (**C** to **E″**) Immunofluorescence on coronal sections of E12.5 cerebellar anlage. The boxed area in (D) is shown on separate channels in (E) to (E″); dashed lines outline the cerebellar anlage; arrows indicate a ventral-to-dorsal extension of Sox14^+^ cells; arrowheads denote Foxp2^+^/Tfap2b^+^/Sox14^−^ cells, presumably nascent PCs, arising from the VZ. (**F**) Illustration showing the section planes. Scale bars, 100 μm.

Prior studies have demonstrated an essential role of *Ptf1a* in the production of GABAergic cerebellar neurons (*23*). Our computational analysis placed *Ptf1a* at the beginning of the gene cascade leading to the generation of PCs (Fig. 2K). We found that the expression of *Lhx1/5*, *Tfap2b*, and *Olig2*, which constitute the gene cascade downstream of *Ptf1a*, was absent from C2 in the E12.5 mouse cerebellum without *Ptf1a* (Fig. 3, A to B′, and fig. S4). Notably, the *Tlx3* expression, which normally demarcates the C3 area, was expanded into the C2 domain (Fig. 3, A to B′, and fig. S4), demonstrating that C2 cells are respecified to a C3 fate in the absence of *Ptf1a* as described previously (*20*). Although *Lhx1/5* and *Tfap2b* are expressed in both C2 and C4, their expression in C4 cells was unaffected in the *Ptf1a*-deficient cerebellum, indicating that C2 and C4 cells are differentially regulated. Therefore, the in vivo studies validate our single-cell trajectory inference and demonstrate the critical role of *Ptf1a* in the specification of the C2 cell fate.

In UMAP embeddings, cells are arranged according to the transcriptome similarity, but not necessarily to the lineage relationship. We postulated that lineage-unrelated clusters could be detected by scrutinizing their temporal appearance along a trajectory. Although the Mes.Isl1 cluster, which represents mesencephalon-derived *Isl1*-expressing cells (*4*), resides at the end of the C3 trajectory, it is abundant and separated from the C3 branch at E10.5 (Fig. 2, A and B), indicating that Mes.Isl1 and C3 cells are lineage unrelated. Prekop *et al.* reported that *Sox14* marks a subset of GABAergic projection neurons in CNs (*24*). However, the origin of these neurons, denoted as CN.gaba, remains undetermined. Notably, CN.gaba cells first appear at E11.5, but they are separated from the C4 and C2 trajectories at this stage (Fig. 2B), suggesting that CN.gaba cells have a different origin from that of C2 or C4 cells. Sox14^+^ cells were initially found in the lateral part of the cerebellar anlage, extending from the basal plate of r1 at E12.5 (Fig. 3C). Near the cerebellar VZ, newly born neurons, presumably nascent PCs, were positive for both Foxp2 and Tfap2b, but negative for Sox14 (Fig. 3, D to E″). Therefore, our data suggest that Sox14^+^ inhibitory projection neurons of CNs originate from the basal plate of r1, rather than the cerebellar VZ.

### Ephemeral Atoh1-expressing cells destined for the Atoh1 lineage from the cerebellar VZ

The *Atoh1* lineage produces diverse cerebellar cell types, including CN neurons, GCs, and unipolar brush cells (*3*, *25*, *26*). Our data suggest that the early *Atoh1*-expressing cells produce early-born CN neurons (CN.early) before E12.5 and then late-born (CN.late) between E12.5 and E13.5 (Fig. 2, A to C). *Lhx2* is expressed in CN.early cells, whereas *Pax6*, *Olig2*, and *Neurod2* are expressed in CN.late cells (fig. S5A). Lhx2^+^ cells occupy the anterior part of the nuclear transitory zone, whereas Pax6^+^, Olig2^+^, and Neurod2^+^ cells are present in the posterior part of the nuclear transitory zone at E13.5 (fig. S5, B and C). At E15.5, the Lhx2^+^ cells are positive for tyrosine hydroxylase (Th), which marks the isthmic nuclei as shown previously through genetic fate mapping with *Fgf8*-*CreER* mice (fig. S5D) (*4*). Pax6 and Olig2 differentially mark in the prospective medial (fastigial) and lateral (interposed and dentate) CNs (*4*, *27*, *28*). Therefore, CN.early likely represent isthmic nuclei neurons, whereas CN.late are cerebellar glutamatergic nuclear neurons.

In contrast to the conventional view that GABAergic and glutamatergic neurons arise from spatially distinct germinal zones (*2*), our trajectory analysis suggested that the same group of NPCs gives rise to the *Atoh1* and *Ascl1* branches between E10.5 and E13.5 (Fig. 2, A to C). Sporadic *Atoh1*^+^ and *Ascl1*^+^ cells were found in proliferative NPCs, NPC.M (NPCs in the M phase), and NPC.S (NPCs in the S phase) (fig. S5E). To evaluate codependency and mutual exclusivity for *Atoh1*, *Ascl1*, and other genes in proliferative NPCs, we calculated the codependency index (CDI) and exclusively expressed index (EEI) (*29*). We found that *Atoh1* and *Ascl1* were expressed in a mutually exclusive manner among proliferative NPCs (fig. S5, F and G, and data S3). *Ascl1* was coexpressed with *Ptf1a* and *Neurog2*, whereas *Atoh1* was with *Olig3* and *Msx1* (fig. S5, F and G, and data S3). The distinct coexpression pattern indicates that the sporadic expression of *Atoh1* and *Ascl1* in NPCs is unlikely technical noise of scRNA-seq but reflects a transient expression of these genes and the associated developmental program.

**Fig. 4.**
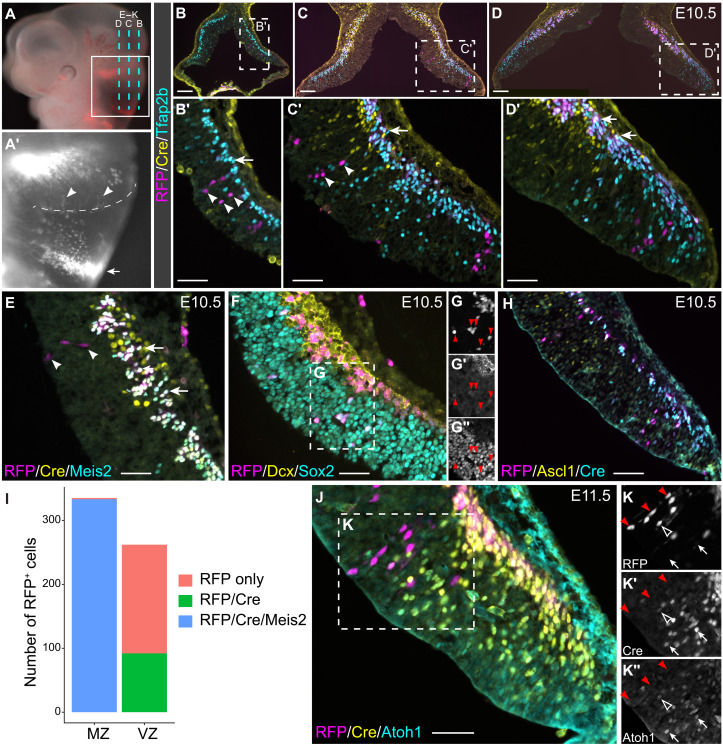
*Atoh1*-expressing cells arising from the cerebellar VZ. (**A** and **A′**) tdTomato fluorescence in whole-mount *Atoh1-Cre; R26*^*RFP*/+^ mouse embryos at E10.5. The boxed area is enlarged in (A′); the dashed lines indicate the section plane of (B) to (D′) and (E) to (K) in (A) and the VZ-roof plate border in (A′); arrowheads indicate RFP-labeled cells in the VZ. (**B** to **H**) Immunofluorescence of E10.5 *Atoh1-Cre; R26*^*RFP*/+^ cerebella. Arrowheads indicate radially oriented RFP^+^/Cre^−^ cells; arrows denote RFP^+^/Cre^+^ cells in the subpial stream. The boxed area in (F) is shown in separate channels (G to G″). (**I**) Histogram of quantification of RFP^+^ cells in the VZ and the mantle zone (MZ). (**J** to **K**) Immunofluorescence of E11.5 *Atoh1-Cre;R26*^*RFP*/+^ cerebella. Red arrowheads indicate RFP-only cells; empty arrowheads show RFP^+^/Cre^+^/Atoh1^+^ cells; arrows denote Cre^+^/Atoh1^+^ cells. Scale bars, 100 μm (B to D and H) and 50 μm (B′ to D′, E, F, and J).

To investigate the generation of the *Atoh1* lineage, we performed genetic fate mapping using an *Atoh1-Cre* transgene (*30*) and the Ai14 Cre-reporter mouse line, denoted as *R26^RFP^*, which expresses red fluorescent protein (RFP; tdTomato) upon Cre-mediated recombination (*31*). RFP-labeled descendants of the *Atoh1* lineage were found in the subpial stream (*32*), the mantle zone underneath the pial surface in *Atoh1-Cre;R26^RFP/+^* embryos at E10.5 and E11.5 (Fig. 4, A to D′). Almost all RFP^+^ cells in the subpial stream were positive for Cre and Meis2 (Fig. 4, B to D′ and I). Unexpectedly, radially oriented RFP^+^ cells were found in the dorsal and anterior parts of the cerebellar VZ in addition to the RL (Fig. 4, B to E). These RFP^+^ cells were positive for Sox2, but not Dcx, indicating that they were progenitors (Fig. 4, F to G″). In contrast to the RFP^+^ cells in the subpial stream, only 35.1% of the RFP^+^ cells in the VZ were positive for Cre (Fig. 4, B to D′ and I). These findings suggest that some NPCs in the cerebellar VZ transiently express Cre, resulting in sporadic, but permanent, RFP labeling of their progeny. The fact that almost all RFP^+^ cells in the subpial stream were positive for Meis2 and Cre suggests that cerebellar VZ progenitors with ephemeral *Atoh1* expression are committed to the *Atoh1* lineage. In support of this notion, Cre^+^ or RFP^+^ cells were negative for Tfap2b and Ascl1, which mark precursors of GABAergic neurons (Fig. 4, B to D′ and H). The expression pattern of RFP and Cre in the cerebellar VZ was undetected in the midbrain and telencephalon of *Atoh1-Cre;R26^RFP/+^* or in the cerebellum of *R26^RFP/+^* embryos. Furthermore, immunostaining showed that Cre and Atoh1 were colocalized in the E11.5 and E12.5 cerebellum (Fig. 4, J to K′), demonstrating that the *Atoh1-Cre* transgene faithfully mimics the endogenous *Atoh1* expression in the cerebellum. Therefore, our scRNA-seq and lineage tracing analyses show that the cerebellar VZ contains transient *Atoh1*-expressing NPCs that are committed to the *Atoh1* lineage between E10.5 and E13.5.

### Single-cell analysis of chromatin accessibility of the embryonic mouse cerebellum

**Fig. 5.**
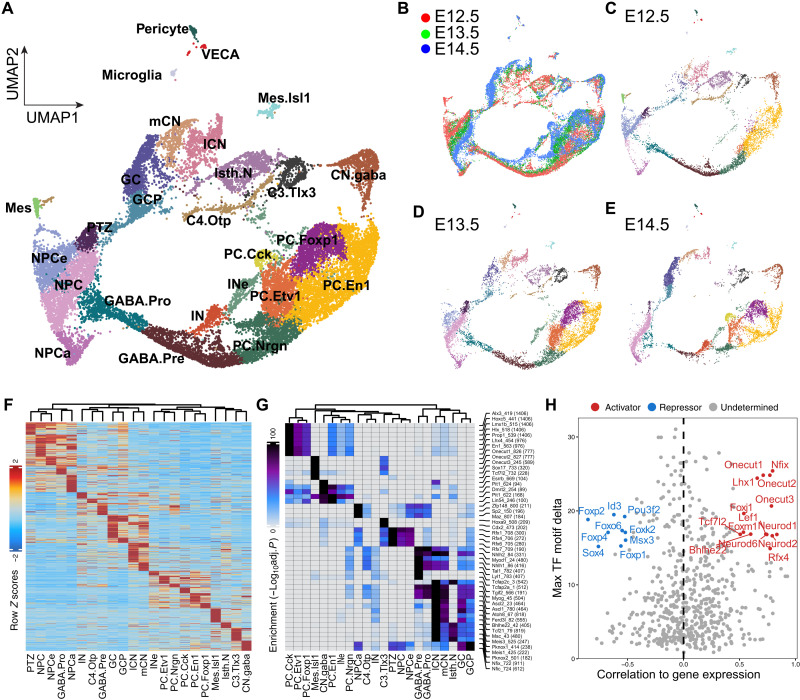
Studying the chromatin landscape in the embryonic mouse cerebellum through snATAC-seq. (**A** to **E**) UMAP projection of snATAC-seq cells. Colors indicate stages in (B) or cell types in the others. (**F** and **G**) Heatmap showing cluster-specific open chromatin regions (F) and the associated TF binding motifs (G). (**H**) Scatterplot showing the correlation between the expression of TFs and their binding motifs in the accessible chromatin region. Putative transcriptional activators and repressors are shown in red and blue, respectively.

To identify CREs and quantify their dynamic activity during early cerebellar development, we performed snATAC-seq of the mouse cerebellum at E12.5, E13.5, and E14.5. Fragment size distribution and transcription start site enrichment demonstrated the high quality of our data (fig. S6, A to D). After stringent selection, we obtained a total of 31,144 high-quality cells, which were clustered into 26 groups (Fig. 5, A to E, and fig. S6E). We determined fixed-width peaks [501 base pairs (bp)] on aggregate single cells of the individual cell group and merged nonoverlapping peaks to a union set of 401,835 peaks, which span over 200 Mb or 10.7% of the reference mouse genome.

To determine the identity of ATAC cell clusters, we calculated “gene activity scores” to estimate gene expression based on local accessibility of the gene body and promoter. We assigned identities according to the activity of known cell-specific markers: *Foxp2* and *Rora* for PCs, *Ptf1a* for GABAergic progenitors (GABA.Pro), *Sox14* for GABAergic CN projection neurons (CN.gaba), *Pax2* for IN, *Meis2* for glutamatergic CN neurons, *Pax6* and *Atoh1* for GCs and GCPs, respectively, and *Id3* for NPCs (fig. S7A). Differential analyses identified cluster-specific markers and confirmed our identity assignment (fig. S7B and data S4). To systematically assess the cell identity, we integrated our in-house E12.5 to E14.5 RNA and ATAC cells, revealing strong concordance between the two modalities—most ATAC cell clusters had one-to-one mapping to the RNA cell clusters (fig. S7C). The integration resulted in a more accurate estimation of gene expression in each ATAC cell (fig. S7, D and E).

Focusing on the E12.5 data, which were sufficient to reconstruct most of the trajectories of Carter’s E10.5 to E13.5 data, we showed that the global structures revealed by UMAP embedding for RNA and ATAC cells were remarkably conserved (fig. S8A). When the scVelo-inferred latent time of the RNA cell was transferred to its nearest ATAC neighbors, we observed a smooth continuum of latent time in the chromatin manifold (fig. S8B). One notable exception was that only the ATAC analysis correctly placed the PTZ cells between the NPC and the *Atoh1* lineage, although both assays recovered the same set of molecular features of PTZ cells (fig. S8C). The better performance of ATAC than RNA analysis in trajectory inference is likely because the former is unobscured by cell cycle genes, which confound the RNA study of dividing cells. Many PTZ feature genes, such as *Atoh1*, *Cdon*, *Hes1*, *Gdf10*, *Nkd1*, and *Reln*, are expressed in distinct but overlapping patterns in the area encompassing the posterior part of the cerebellar VZ and morphologically defined RL (fig. S8, C to E). To distinguish it from the RL, we refer to this VZ domain as PTZ.

### Examination of cell type–specific accessible chromatin regions and their associated TF-binding motifs

We found that 40.5% of the total ATAC peaks exhibit differential accessibility among cell clusters [a median of 9761 peaks per cluster; false discovery rate (FDR) < 0.01 and log_2_ fold change (FC) ≥ 1; Fig. 5F], demonstrating that different cell types and states have distinctive chromatin landscapes. Motif analysis revealed that the cluster-specific loci are enriched for different TF binding motifs: The *Atoh1* lineage, including CNs, GCs, and GCPs, is enriched for binding motifs of nuclear factor I (NFI) TFs and basic helix-loop-helix TFs, NPCs for regulatory factor X (RFX) TFs, and PCs for homeodomain-containing TFs (Fig. 5G). We compiled a list of genomic features on the basis of published snATAC-seq, bulk chromatin immunoprecipitation, and deoxyribonuclease (DNAse) I hypersensitive site (DNaseHS) sequencing to perform overlapping enrichment testing. As expected, cell type–specific open chromatin regions are largely consistent with those identified by a recent snATAC-seq of mouse cerebella (fig. S7F) (*13*). The ATAC peaks specific for GABAergic progenitors are enriched for DNA sequences bound by Ascl1 and Ptf1a (*33*), whereas those specific for GCs and GCPs are enriched for sequences bound by Atoh1 (fig. S7F) (*34*). Notably, chromatin regions identified by bulk analysis of H3K27ac, and DNaseHS of postnatal mouse cerebella, are notably enriched in GC-specific peaks, likely due to the prevalence of GCs in the postnatal cerebellum (fig. S7F). Collectively, our findings demonstrate that different cerebellar cell types are regulated by distinct regulatory programs.

Next, we applied chromVAR (*35*) to calculate the relative motif activity at single-cell resolution. By correlating chromVAR deviation *z* scores of TF motifs with expression levels of TFs, we systematically deduced positive and negative TFs in the developing cerebellum based on whether their expression was positively or negatively correlated with motif enrichment. We iterated the analysis in each stage and identified a total of 33 putative activators and 16 repressors (Fig. 5H). In support of this categorization, the majority of the TFs in these two groups are known transcriptional activators (29 of 33; 87.9%) or repressors (10 of 16; 62.5%), according to the Gene Ontology Consortium (data S5). As the expression of these factors is closely correlated with the accessible chromatin region with their binding motifs, they may function as pioneer factors (*36*) and play crucial roles in cell fate specification in the developing cerebellum.

### Identification of CREs for cell type–specific transcriptional programs

**Fig. 6.**
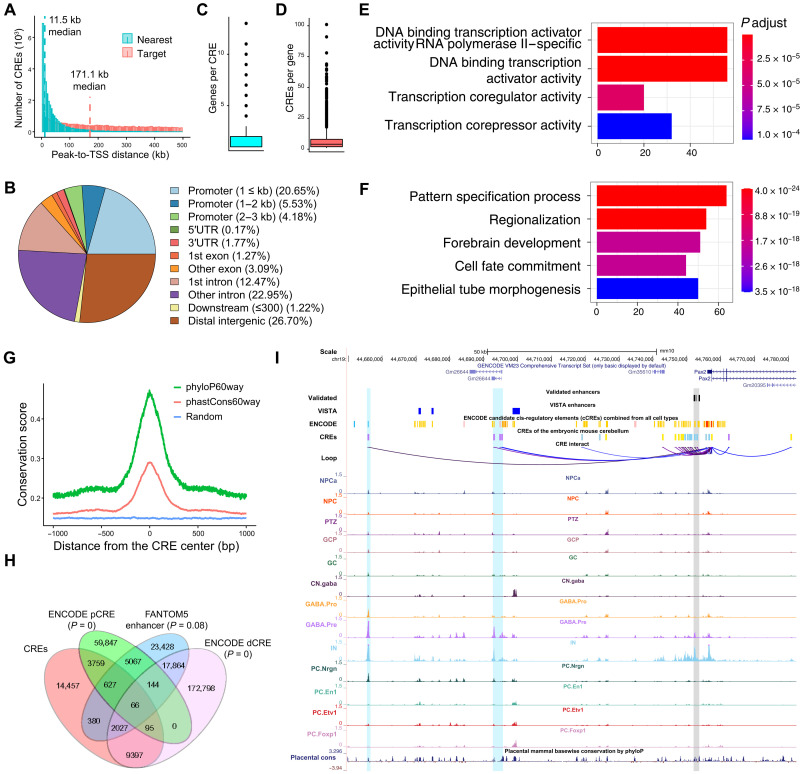
Characterization of CREs in the embryonic mouse cerebellum. (**A**) Histograms comparing the distribution of distances from CREs to the nearest genes versus target genes. (**B**) Pie chart showing the distribution of CREs among genomic features. (**C** and **D**) Box-whisker plots showing the number of genes per CRE (C) and the number of CREs per gene (D). (**E** and **F**) Bar plots showing enrichment of Gene Ontology in molecular function (E) and biological process (F). (**G**) Distribution of the average base pair evolutionary conservation scores using 60-vertebrate genome alignments at regions around CREs and randomly shuffled regions. (**H**) Venn diagram showing overlapping of CREs with proximal (pCRE) and distal elements (dCRE) identified by ENCODE or enhancers by FANTOM5 (*37*, *38*). The percentages indicate the elements unique to each group. (**I**) Genome browser tracks showing linkages of CREs and Pax2. The gray area highlights the CRE that has been validated by transgenic mouse assays; cyan highlights indicate two putative CREs that are specifically activated in GABA.Pre and IN.

To find CREs, we identified chromatin-accessible peaks that correlated with transcription in the combined E12.5 to E14.5 ATAC-RNA joined cell clusters. We iterated the analysis for individual stages and identified a total of 44,329 peak-to-gene pairs with 31,088 unique accessible peaks and 7159 target genes. A total of 69.4% of the identified CRE-target pairs are specific to a particular stage, demonstrating the temporally dynamic activation of most CREs. The median distance between CREs and target promoters is 171.1 kb, 14.9 times greater than that to the closest genes (Fig. 6A). Most CREs are located in the intronic or distal intergenic regions (Fig. 6B), suggesting that the identified CREs are mostly distal elements. While 73.0% of the CREs are assigned to a single gene, the rest are linked to two or more (up to 13; Fig. 6C), showing that some CREs may regulate multiple genes. The median number of CREs per gene is four (Fig. 6D). The top 10 percentile genes with the highest numbers of CREs (>16) are enriched for DNA binding transcription regulator activity and regulation of neural development (Fig. 6, E and F, and data S6). The top eight genes—*Zic5*, *Zic2*, *Pax6*, *Hes5*, *Sox9*, *Lhx1*, *Sox2*, and *Pax3*—are well-known TFs important for cerebellar development. These observations suggest that key TFs acting in early cerebellar development are subjected to complex regulation via numerous CREs in agreement with a recent report (*13*).

Additional lines of evidence support the functional significance of the identified CREs. First, we detected a sharp increase in evolutionary conservation scores at the center of CREs (Fig. 6G). Furthermore, the CREs are significantly overlapped with putative enhancers identified by The Encyclopedia of DNA Elements (ENCODE) consortium and Functional Annotation of the Mammalian Genome Project 5 (FANTOM5) (Fig. 6H) (*37*, *38*). Last, we asked whether our approach recovered the enhancers that had been validated by transgenic mouse assays. We compiled a list of the enhancers of seven genes that are involved in cerebellar development, including *Atoh1*, *Fgf8*, *Kirrel2*, *Pax2*, *Pcp2*, *Ptf1a*, and *Wnt1* (*39*–*45*). All these validated enhancers were recovered by our study (Fig. 6I and fig. S9). Therefore, our integrative scRNA-seq and snATAC-seq analyses provide valuable information on the CREs that control the temporospatial gene expression in the developing cerebellum.

### Reconstruction of GRNs governing cerebellar development

**Fig. 7.**
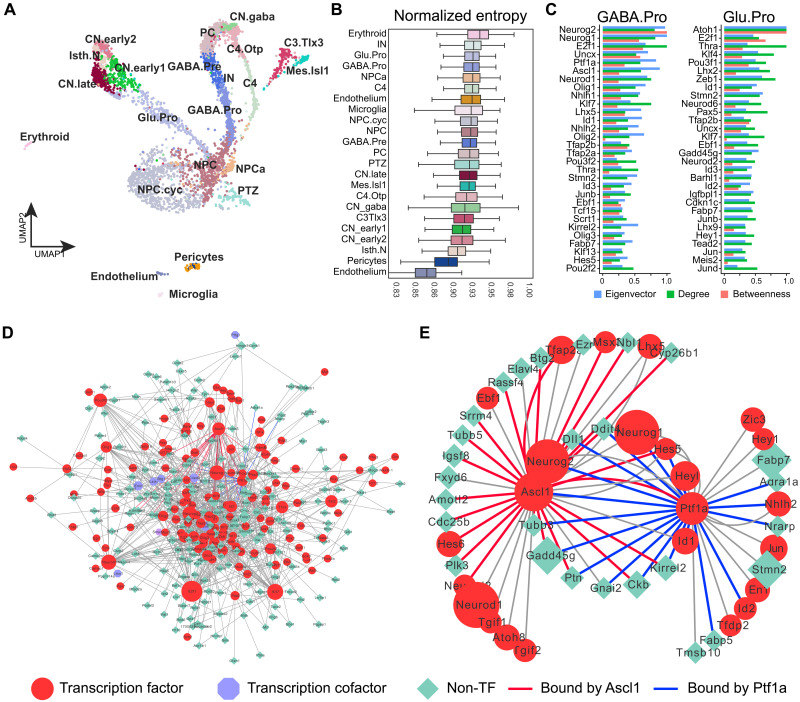
Reconstruction of GRNs of cerebellar cell types and states. (**A**) UMAP projection of in-house E12.5 scRNA-seq. (**B**) Box plot showing the distribution of network entropy scores for each cell cluster. (**C**) Top 30 driver genes by an aggregate ranking of degree centrality, betweenness centrality, and eigenvector centrality in the GRNs of GABA.Pro and Glu.Pro. (**D**) The inferred GRN of GABA.Pro cells. (**E**) Subnetworks of (D) with all targets of Ascl1 and Ptf1a. Links confirmed by chromatin immunoprecipitation sequencing are shown in red (for Ascl1) and blue (for Ptf1a).

We leveraged the newly identified CREs to reverse engineer GRNs by applying CellOracle. For each cell type and state at E12.5 (Fig. 7A), we produced a GRN, which exhibits a scale-free network characteristic for biological networks. The network entropy, indicative of the average undifferentiated state, decreases from progenitors (such as NPCs, GABA.Pro, and Glu.Pro) to postmitotic cells (such as PCs and GCs) as expected (Fig. 7B). We identified key regulators of each cell-specific GRN through network analyses (Fig. 7C, fig. S10, and data S7). Among the predicted top regulators of GABAergic progenitors, *Neurog1/2*, *Ptf1a*, *Ascl1*, *Olig2/3*, *Tfap2a/b*, and *Lhx1/5* have been shown to play crucial roles in the development of GABAergic neurons in the cerebellum (*23*, *46*–*51*). Similarly, in vivo studies have demonstrated the essential role of *Atoh1*, *Neurod2*, *Barhl1*, and *Lhx9* in cerebellar glutamatergic neurons as predicted by our GRN analysis (Fig. 7C) (*52*–*56*). In the GRN of GABAergic progenitors, the majority of the CREs of the inferred targets of Ascl1 and Ptf1a are bound by Ascl1 (76.7%) and Ptf1a (75.0%) according to a prior binding profile study (Fig. 7, D and E) (*33*).

### Studying the molecular regulation of PTZ development via GRN simulations

**Fig. 8.**
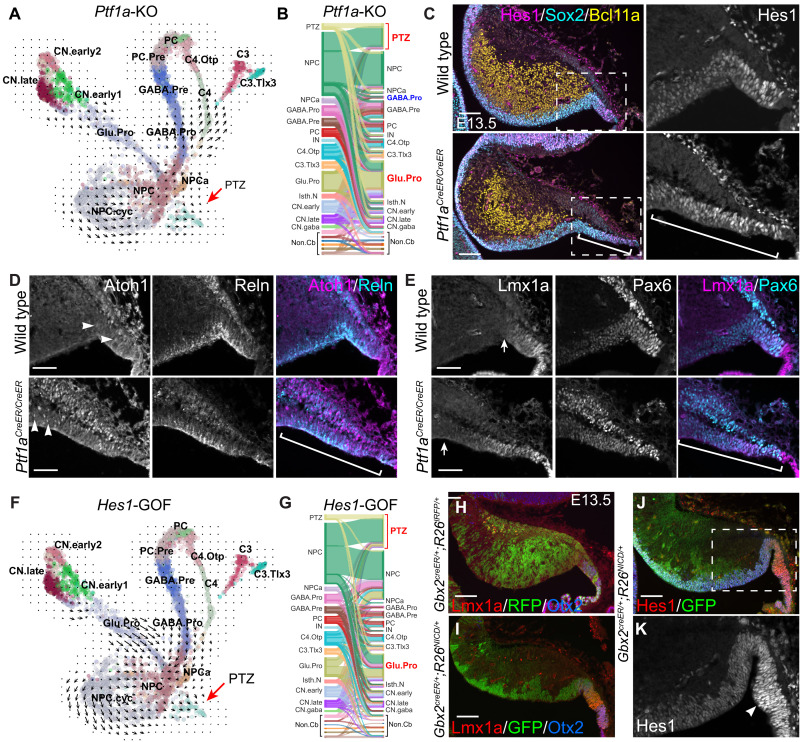
Investigation of the molecular control of PTZ development by GRN simulations and genetic experiments. (**A** and **B**) The vector field graph and Sankey diagram showing simulated cell transition caused by the loss of *Ptf1a*. The arrow and brackets indicate the enlarged PTZ and RL caused by the mutation. (**C** to **E**) Immunofluorescence on sagittal sections of E13.5 cerebella. The boxed areas are enlarged in (D) and (E); the brackets indicate the enlarged PTZ and RL domain, which is marked by *Hes1*, *Reln*, and *Lmx1a*; arrowheads indicate nascent Atoh1^+^ cells (*n* = 3); and arrows denote the anterior limit of Lmx1a expression domain. (**F** and **G**) The vector field graph and Sankey diagram showing the effects of *Hes1* overexpression. (**H** to **K**) Immunofluorescence on cerebellar sagittal sections of E13.5 *Gbx2^CreER/+^;R26^RFP/+^* or *Gbx2^CreER/+^;R26^NICD/+^* mice that were given tamoxifen at E8.5 (*n* = 5). The boxed area in (J) is enlarged in (K); the arrowhead shows robust Hes1 expression in the enlarged PTZ and RL. Scale bars, 100 μm (C and H to J) and 50 μm (D and E).

To evaluate the validity and utility of the GRNs, we applied CellOracle to simulate how TF perturbations affect cell fate decisions. We focused on *Ptf1a* and *Atoh1*, two well-characterized master regulators in cerebellar development. In agreement with mouse genetic studies (*23*, *26*, *52*), GRN simulations predicted that the loss of *Ptf1a* would reduce GABAergic progenitors, whereas the loss of *Atoh1* would deplete glutamatergic progenitors (Fig. 8, A and B, and fig. S11A). Unexpectedly, GRN simulations suggested that the loss of *Ptf1a* would enlarge the PTZ (Fig. 8, A and B). By examining a panel of PTZ markers, including Atoh1, Lmx1a, Hes1, Otx2, Pax6, and Reln, we found that the PTZ domain was notably enlarged in *Ptf1a*-deficient cerebellum at E13.5 (Fig. 8, C to E). In agreement with the previous findings (*18*), GRN simulations showed that the loss of function and GOF of *Lmx1a* would reduce or enlarge, respectively, the PTZ (fig. S11, B and C).

*Hes1* and *Hes5*, which are important for NPC maintenance (*57*), are expressed in the cerebellar VZ, whereas only *Hes1* is expressed in the PTZ (fig. S12, A and B). GRN simulations predicted that the loss of function or GOF of *Hes1* would reduce or enlarge the PTZ, respectively (Fig. 8, F and G, and fig. S12C). To investigate the epistasis between *Hes1* and *Ptf1a* or *Lmx1a*, we performed additional GRN simulations and found that *Hes1* acted downstream to mediate the function of *Ptf1a* and *Lmx1a* on PTZ development (fig. S12D).

*Hes1* is a well-known transcriptional target of Notch signaling (*57*). Upon activation, the Notch intracellular domain (NICD) is released from the cell membrane and translocated into the nucleus to promote *Hes1* transcription (*57*). We predicted that an ectopic expression of *NICD* would induce *Hes1*, resulting in a GOF of *Hes1* and thereby an enlargement of the PTZ. To test our hypothesis, we conditionally expressed *NICD* in the cerebellar VZ by combining *Gbx2^CreER^* (*58*) and *R26^NICD^*, in which *NICD-ires-GFP* is expressed from the *Rosa26* locus upon Cre-mediated recombination (*59*). After tamoxifen administration between E8.5 and E10.5, we detected descendants of the *Gbx2* lineage throughout the cerebellum in *Gbx2^CreER/+^;R26^RFP/+^* embryos at E13.5 (Fig. 8H). By contrast, *NICD*-expressing (*NICD^+^*) cells, which were marked by green fluorescent protein (GFP) immunoreactivity, were restricted to the VZ, PTZ, and RL in *Gbx2^CreER/+^;R26^NICD/+^* embryos (Fig. 8, I and J). As expected, robust *Hes1* expression was detected in *NICD^+^* cells, particularly in the PTZ (Fig. 8, J and K). The PTZ was markedly enlarged in *NICD*-GOF embryos at E13.5 (Fig. 8, H to K). These results reveal a crucial role of *Hes1* in PTZ development and demonstrate the efficacy of GRN stimulations to predict the outcome of TF perturbations.

### Growth of the posterior cerebellar vermis depends on de novo induction of the Atoh1 lineage

**Fig. 9.**
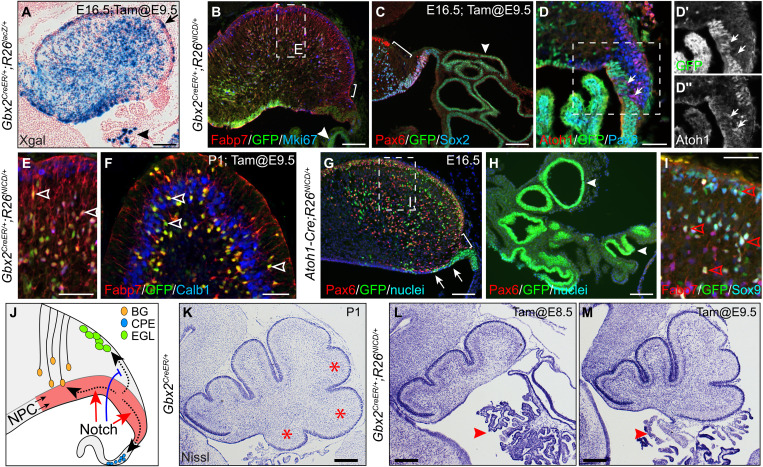
Specific loss of the posterior cerebellar vermis due to the abnormal cell fate decision in the PTZ and RL. (**A**) Xgal histochemistry showing the ubiquitous distribution of *Gbx2*-expressing cells labeled at E9.5 in the E16.5 cerebellum (*n* > 3). The arrow indicates Xgal^+^ cells in the EGL; the arrowhead shows a few Xgal^+^ cells in the choroid plexus (CP). (**B** to **F**) Immunofluorescence on cerebellar sagittal sections of *Gbx2^CreER/+^;R26^NICD/+^* mice that were given tamoxifen at E10.5 (*n* = 7). The brackets indicate the discontinuous EGL; arrowheads show the accumulated NICD^+^ cells in the enlarged CP; arrows show Atoh1^+^ cells devoid of NICD; and empty arrowheads denote NICD^+^ Bergmann glia. (**G** to **I**) Immunofluorescence on cerebellar sagittal sections of *Atoh1-Cre;R26^NICD/+^* mice at E16.5 (*n* = 3). Arrowheads show NICD^+^ cells accumulated in the expanded CP; arrows indicate *NICD*^+^ cells in the VZ outside the PTZ; and the empty arrowheads denote NICD^+^ Bergmann glia. (**J**) Illustration of the transitioning progenitor cells in the region encompassing the PTZ and RL having three-prong trajectories (dashed arrows) that are promoted (red) or inhibited by Notch signaling. (**K** to **M**) Nissl histology of the sagittal section of P1 cerebellum. The arrowheads indicate the CP that is greatly expanded. Note the progressive loss of posterior cerebellar vermis [asterisks in (K)] when NICD was induced in *Gbx2^CreER/+^;R26^NICD/+^* at E8.5 and E9.5. Scale bars, 100 μm (A to C, G, and H), 50 μm (D to F and I), and 250 μm (K to M).

In contrast to the sparse contribution of progenies of *Gbx2*-expressing cells labeled at E9.5 to the choroid plexus in the control, abundant *NICD*^+^ cells were found in the exceedingly enlarged choroid plexus in E16.5 *Gbx2^CreER/+^;R26^NICD/+^* embryos (Fig. 9, A to C). Although RFP-labeled descendants of the *Gbx2* lineage abundantly contribute to the external granular layer (EGL), few *NICD*^+^ cells were detected in the EGL (Fig. 9, B and C). At the RL, the expression of NICD (GFP) and Atoh1 was mutually exclusive, indicating a cell-autonomous inhibition of *Atoh1* by NICD (Fig. 9, D to D″). A gap was detected between the EGL and RL in E16.5 *Gbx2^creER/+^;R26^NICD/+^* embryos that were given tamoxifen at E8.5 or E9.5, indicating a disruption of the ongoing induction of *Atoh1* lineage in the presence of Notch signaling (Fig. 9, B and C). In the cerebellar cortex, almost all *NICD*^+^ cells displayed the morphology and molecular features characteristic for Bergmann glia, including the expression of Fabp7, Mki67, Sox2, and Sox9 (*60*), indicating that Notch promotes Bergmann glia generation (Fig. 9, E and F).

As *NICD* was induced broadly in the cerebellar VZ of *Gbx2^creERR/+^;R26^NICD/+^* cerebella, it was unclear whether the NICD-induced Bergmann glia were derived from progenitors fated to the EGL. To address this question, we repeated the *NICD*-GOF experiment with *Atoh1-Cre*. As expected, RFP^+^ fate-mapped *Atoh1* progenies were predominantly found in the EGL of *Atoh1-Cre;R26^RFP/+^* cerebellum at E18.5 (fig. S13, A and A′). Although a few RFP^+^ cells were present in the choroid plexus, none became Bergmann glia (fig. S13, A and A″). When *NICD* was induced in the *Atoh1* lineage, many *NICD*^+^ cells were detected in GCs and CN neurons of *Atoh1-Cre;R26^NICD/+^* embryos (Fig. 9G and fig. S13, B to C″), showing that *Atoh1* lineage derivatives can tolerate persistent NICD expression. Therefore, the lack of *NICD^+^* cells in the EGL of *Gbx2^creER/+^;R26^NICD/+^* embryos likely results from the abnormal cell fate specification rather than a selective cell loss. As found in *Gbx2^creER/+^;R26^NICD/+^*, the EGL at the RL was much thinner and irregular, and nascent Atoh1^+^ cells were mutually exclusive for NICD^+^ cells in the *Atoh1-Cre;R26^NICD/+^* cerebellum, confirming the inhibitory role of Notch in replenishing the *Atoh1* lineage (fig. S13, D to D″). In contrast to the almost exclusive contribution of RFP^+^ cells to glutamatergic neurons in *Atoh1-Cre;R26^RFP/+^* embryos, abundant *NICD^+^* cells were found in the markedly expanded choroid plexus and Bergmann glia in *Atoh1-Cre;R26^NICD/+^* embryos (Fig. 9, G to I). Therefore, the forced expression of *NICD* in nascent *Atoh1*-expressing cells respecifies the *Atoh1* lineage to form choroid plexus epithelium and Bergmann glia.

Last, we repeated *NICD*-GOF experiments using *Wnt1-CerER* transgenic mice. *Wnt1*-expressing cells contribute to the EGL, choroid plexus, and Bergmann glia (*61*). In *Wnt1-CreER;R26^NICD/+^* embryos that received tamoxifen at E8.5, *NICD^+^* cells accumulated in the greatly enlarged choroid plexus, but were hardly found in the EGL (fig. S13, E and F). We detected abundant *NICD^+^* cells that coexpressed Fabp7 and Sox9 and displayed typical Bergmann glial morphology in the cerebellar cortex of *Wnt1-CreER;R26^NICD/+^* embryos (fig. S13, E and F). Collectively, our data show that the activation of Notch signaling promotes progenitors at the RL to form choroid plexus epithelium and Bergmann glia at the expense of the *Atoh1* lineage (Fig. 9J).

In *Gbx2^CreER/+^;R26^NICD/+^* mice that were given tamoxifen between E8.5 and E9.5, the posterior part of the cerebellum was truncated while the choroid plexus was greatly expanded (Fig. 9, K to M). A similar phenotype was found in *Wnt1-CreER;R26^NICD/+^* cerebella at P15 (fig. S13, G to I). The cerebellar hypoplasia was more severe near the medial than the lateral region of the cerebellar vermis (fig. S13, H and I). Our results demonstrate that sustained replenishment of de novo *Atoh1* lineage is crucial for the growth of the posterior part of the cerebellar vermis.

### Development of the PTZ may underlie the pathogenesis of DWM

**Fig. 10.**
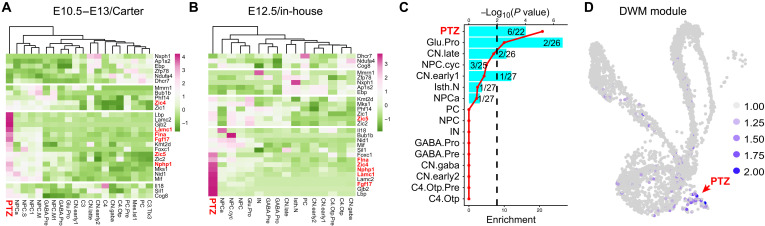
Enrichment of DWM genes the PTZ. (**A** and **B**) Heatmap showing average expression of DWM genes in different cerebellar cell types in Carter’s E10.5 to E13.5 (A) and in-house E12.5 (B) scRNA-seq. Note the elevated expression of DWM genes in PTZ cells (in red). (**C**) Histogram showing enrichment of DWM genes in PTZ cell markers. Line and dots show *P* values (Fisher’s exact test); black dashed line indicates the cutoff for significance at *P* = 0.01; numbers show cell-specific markers that are overlapped or nonoverlapped with DWM genes. (**D**) Expression of module scores of aggregated expression of DWM genes [shown in red in (A) and (B)] in the in-house E12.5 scRNA-seq. The arrow indicates the elevated expression in the PTZ.

DWM represents the most common congenital human cerebellar defects, which are defined by cerebellar vermis hypoplasia and enlarged posterior fossa (*62*, *63*). To investigate whether PTZ development is involved in DWM, we examined the expression of genes associated with DWM among different cerebellar cell types. DWM candidate genes were enriched in the PTZ in both Carter’s and our in-house scRNA-seq data (Fig. 10, A and B). Differential expression and gene set enrichment analysis showed that PTZ markers were significantly enriched for DWM genes, including *Fgf17*, *Flna*, *Lamc1*, *Nphp1*, *Zic4*, and *Zic5* (Fig. 10C). The aggregated expression of these six candidate genes was restricted to PTZ cells (Fig. 10D). Collectively, our data suggest that abnormal development of the PTZ underlie the pathogenesis of DWM.

## DISCUSSION

### Integrated analyses of transcriptome and chromatin accessibility at single-cell resolution

Through scRNA-seq trajectory analyses, we have reconstructed the developmental dynamics of the entire cerebellar primordium and in individual lineages during mouse embryogenesis. We confirm the four different cellular populations originating from distinct compartments of the cerebellar VZ and infer gene expression cascades along their developmental trajectories. Our results provide new information on the molecular mechanisms that create the heterogeneity of glutamatergic (see next section) and GABAergic neurons. We suggest that Sox14^+^ inhibitory projection neurons of the CNs may originate outside the cerebellar primordium, probably from the basal plate of the neural tube. Together with our previous finding of mesencephalon-derived Isl1^+^ cells (*4*), our results suggest that the migration of cells that originated outside the cerebellar anlage may represent an important mechanism to increase the cellular diversity of the cerebellum.

By applying paired single-cell RNA-ATAC analysis, we have systematically studied the regulome that governs cell state transition and lineage commitment in the developing mouse cerebellum. We identify 31,088 candidate CREs with their targets and predicted TFs that act through these candidate CREs. The reference maps of CREs for the mouse cerebellum will not only help to understand the mechanism of gene regulation in different cell types but also enable targeting and purifying of specific cell types. Built on the newly identified CREs, we have reverse engineered GRNs for individual cell types and demonstrate the validity and utility of the GRNs. Our study demonstrates that GRN simulations can be used to evaluate how genetic perturbations affect cell fate decisions, informing strategies for cell reprograming to produce specific cerebellar cell types in vitro. In addition, GRN simulations can be extended to study multiple genes to evaluate genetic redundancy and epistasis. To this end, we strive to make our data broadly available to the community (see “Data and materials availability” statement).

Despite the notable capability of GRN analysis, the current method has limitations and dependencies. First, the accuracy of the predicted TFs based on peak-gene pairs depends on the availability of high-quality binding motifs. Second, when simulating TF perturbation, CellOracle predicts the directionality of the fate changes in affected cells expressing the particular TF, but not the long-term consequences. Furthermore, only TFs with relatively high variability and expression values are available for perturbation simulations.

### The cerebellar VZ contains ephemeral Atoh1-expressing progenitors destined to the Atoh1 lineage

Past studies have demonstrated the extraordinary cellular diversity of *Atoh1* derivatives in the cerebellum (*3*, *25*, *26*). *Atoh1*-expressing cells arising from the RL between E9.5 and E12.5 produce CN neurons (*3*). It has been shown that the earliest neural precursor cells exit the cell cycle from the cerebellar VZ at E10.25 and contribute to CN neurons (*64*, *65*). These early-born CN neurons express *Irx3*, *Meis2*, and *Lhx2/9*, and they undergo radial migration to reach the nuclear transitory zone (*64*). The relationship between the early-born CN neurons from the VZ and the *Atoh1* lineage has never been determined. Here, we provide evidence that the early-born CN neurons present by E10.5 are probably derived from cerebellar NPCs with ephemeral *Atoh1* expression (Fig. 2, A and B). Therefore, our observations reconcile the previous findings on the origin of CN neurons. Green *et al.* showed that some *Atoh1*-expressing cells are generated at the isthmus, independent of the RL, producing isthmic nuclei (*66*). Using trajectory analysis, we infer that the early-born *Atoh1* lineage gives rise to the isthmic nuclei. However, intersectional genetic fate mapping or integrated lineage tracing and scRNA-seq are required to determine the cell fate of the transient *Atoh1*-expressing cells from the cerebellar VZ.

NPCs, including those in the anterior part of the cerebellar VZ, express Cre that is driven by an *Atoh1* enhancer (Fig. 4). In contrast to the relative abundance of Cre^+^ cells, RFP^+^ fate-mapped cells arising from the VZ are rare and mostly negative for Cre in *Atoh1-cre;R26^RFP/+^* embryos, suggesting that only a small percentage of Cre^+^ NPCs is labeled because of the ephemerality of Cre expression. Almost all RFP^+^ cells are negative for Tfap2b and Ascl1 and become positive for Meis2 and Cre when they reach the mantle zone (Fig. 4), indicating that the NPCs that transiently express *Atoh1* are committed to the *Atoh1* lineage. Basic helix-loop-helix TFs, such as Ascl1, Hes1, and Olig2, are expressed in an oscillating manner to stabilize the stemness of NPCs (*57*, *67*). Future studies should determine whether the oscillatory control of *Atoh1* specifies and sustains CN progenitors in the cerebellar VZ.

### The PTZ contains multipotent progenitor cells

The identity of the stem cell reservoir for the continuous production of *Atoh1*-expressing cells at the RL has yet to be identified. On the basis of the patterns of gene expression and cell proliferation, Yeung *et al.* speculated that the stem cells fated to the *Atoh1* lineage reside in the interior side of the RL, and they express both *Wls* and *Pax6* (*68*). However, the expression patterns of classic RL markers, such as *Wls*, *Pax6*, *Lmx1a*, and *Wnt1*, change over time in the developing RL (*18*, *61*, *68*, *69*), and the progenitor domain marked by *Pax6* and *Wls* appears larger than the morphologically defined RL (*32*, *68*). In a previous scRNA-seq study (*4*), we identified an inseparable NPC cluster that has the potential to form both the *Atoh1* lineage and choroid plexus epithelium and mapped that cell group to the posterior end of the cerebellar VZ and RL. In the current study, we identified the same NPC cluster by integrated single-cell RNA and ATAC analyses. To distinguish it from the morphologically defined RL, we named the VZ region occupied by this cell group as PTZ as we postulated that cerebellar NPCs may be recruited into the PTZ and transition into progenitors destined for glutamatergic neurons and choroid plexus epithelium.

We show that *Hes1* is expressed in the PTZ, which specifically lacks *Hes5* (fig. S11B). Through in silico and in vivo studies, we demonstrate that the elevated expression of *Hes1*, as a result of the loss of *Ptf1a* or forced expression of *NICD*, enlarges the PTZ and RL, suggesting that *Hes1* is important to recruit NPCs to the RL through the PTZ (Fig. 8). Notch activation promotes choroid plexus epithelium but inhibits *Atoh1* expression (Fig. 9). The latter is in agreement with the previous report that Notch activation inhibits *Atoh1* expression by antagonizing bone morphogenetic protein signaling (*70*). Somewhat unexpectedly, we found that the forced expression of *NICD* induced Bergmann glia not only from *Gbx2*-expressing cells but also from the *Atoh1* lineage, demonstrating that Notch activation respecifies nascent *Atoh1* cells to form Bergmann glia and choroid plexus. Although it is well known that Notch signaling is important for Bergmann glia differentiation (*71*), our results uncover the involvement of Notch signaling in the induction of Bergmann glia. We have previously shown that an FGF-ERK-ETV axis of the mitogen-activated protein kinase (MAPK) pathway is essential for the transition of cerebellar radial glia to Bergmann glia (*60*, *72*). How Notch interacts with the FGF-ERK-ETV axis to induce Bergmann glia awaits to be examined. Prior studies have shown that nascent Bergmann glia arise from a VZ domain, the so-called peritrigonal glial matrix (*73*), overlapping with the PTZ (*72*, *74*, *75*). As one of the Bergmann glia markers, *Gdf10* is expressed within the PTZ (fig. S8C). Together, our results suggest that the PTZ represents a highly dynamic progenitor zone, where the balance of cell fate commitments is regulated by Notch signaling, in part through Hes1 (Fig. 9J).

Although PTZ cells are identified as a single-cell cluster, several PTZ markers display spatial gene expression gradients along the anteroposterior axis in the PTZ and RL (Fig. 8, D and E, and fig. S8). Therefore, the PTZ cell cluster may contain progenitors of different cell states. Further studies, especially high-resolution spatial expression and lineage tracing, are required to resolve the potentially different cell states within the PTZ and its relationship with the traditionally defined RL.

### The maintenance and differentiation of PTZ cells control the expansion of the posterior cerebellar vermis

Disproportionate reduction in the posterior cerebellum is a hallmark of many human cerebellar congenital defects, including DWM and cerebellar vermis hypoplasia (*5*). It was suggested that spatiotemporal expansion of the RL might be specific to humans, raising the question of whether rodents are suitable to study cerebellar malformations in humans (*5*, *63*). In the present study, we demonstrate that the recruitment of NPCs into the PTZ to replenish the *Atoh1* lineage is essential for the enlargement of the posterior cerebellar vermis. The *NICD*-GOF mice display features that clinically define DWM, including a hypoplastic, upwardly rotated vermis, an enlarged fourth ventricle, and an enlarged posterior fossa (*63*). Furthermore, mouse orthologs of the human genes that have been implicated in DWM are significantly enriched in the PTZ in the embryonic mouse cerebellum. Therefore, abnormalities in the cell fate decisions of PTZ cells may contribute to the DWM pathogenesis. In support of this notion, the loss of *Foxc1*, a bona fide DWM-causing gene, disrupts cell proliferation in cerebellar VZ and the production of Bergman glia (*62*, *76*). Future studies of the maintenance and differentiation of the PTZ in mice should shed light on pathological mechanisms underlying human cerebellar birth defects.

## MATERIALS AND METHODS

### Mouse and tissue preparation

All procedures involving animals were approved by the Animal Care Committee at the University of Connecticut Health Center (protocol #101849–0621) and complied with national and state laws and policies. All mouse strains were maintained on CD1 outbred genetic background. Noon of the day on which a vaginal plug was detected was designated as E0.5 in the staging of embryos. Embryonic mouse brains were dissected in ice-cold phosphate-buffered saline and were fixed in 4% paraformaldehyde between 40 min and overnight. Brains were cryoprotected, frozen in Optimal cutting temperature compound (Sakura Finetek), and sectioned with cryostat microtome (Leica).

Generation and characterization of the *Atoh1-Cre* [B6.Cg-Tg(Atoh1-cre)1Bfri/J, #011104] (*30*), *Gbx2^creER^* [*Gbx2^tm1.1(cre/ERT2)Jyhl^*/J, #022135], *Ptf1a^CreER^* [*Ptf1a^tm2(cre/ESR1)Cvw^*/J, #019378], *R26R^RFP^* [*B6.Cg-Gt(ROSA)26Sor^tm9(CAG-tdTomato)Hze^*/J, #007909], *R26^NICD^* [*Gt(ROSA)26Sor^tm1(Notch1)Dam^*/J, #008159], and *Wnt1-CreER* [Tg(Wnt1-cre/ERT)1Alj/J, #008851] alleles have been described. Primer sequences for polymerase chain reaction genotyping and protocols are described on the JAX Mice website (www.jax.org).

### In situ hybridization and immunohistochemistry

Standard protocols were used for Xgal histochemistry, immunofluorescence, and in situ hybridization as described previously (*58*). Detailed protocols are available on the Li Laboratory website (http://lilab.uchc.edu/protocols/index.html). Primary and secondary antibodies used in the study are listed in data S8. Standard in vitro transcription methods using T7 polymerase (Roche) and digoxigenin–UTP (uridine triphosphate) RNA labeling mix (Roche) were used to produce antisense riboprobes. Images were collected on a Zeiss Axio Imager M1 microscope and processed using Photoshop or Fiji software.

### Cell counting

Nuclear segmentation was performed on the basis of 4′,6-diamidino-2-phenylindole staining using the Fiji Stardist plugin. The Fiji Annotater plugin was used to correct Stardist-produced segmentations and to identify markers associated with the segmented nuclei, manually for RFP and by using thresholding for Cre- and Meis2-stained nuclei.

### scRNA-seq and data processing

Single-cell preparation was performed as described (*4*), and papain (Worthington Biochemical) instead of Accumax was used for tissue digestion. Sequencing libraries of E12.5 and E14.5 cerebella were generated with Chromium v2 and v3 chemistry, respectively. For Carter’s (PRJEB23051) and Vladoiu’s (GSE118068) dataset, BAM files were downloaded from the European Nucleotide Archive and were converted to FASTQ files using the bamtofastq tool (10X Genomics). RNA-seq reads were aligned to the mm10 reference genome and quantified using the cellranger count function (CellRanger v3.1). Velocyto (v0.17.17) was used to obtain splicing-specific count data for RNA velocity analysis. Count data were processed with the Seurat R package (*77*). From the SeuratWrapper package, the FastMNN function was used to integrate datasets. Differential gene expression analysis was performed with the wilcoxauc function from the presto package. We removed seven cell clusters (a total of 3757 cells) that were presumably derived from noncerebellar tissues included during dissection, as they were absent from Carter’s and in-house data. The numbers of cells used in this study are summarized in table S1.

### PAGA, scVelo, and CellRank analyses

To perform trajectory inference using the Scanpy (Single Cell Analysis in Python; v1.5.0) toolkit (*78*), we converted the Seurat object to AnnData using custom R scripts. PAGA based on embryonic stages was computed and projected using a ForceAtlas2 or hierarchical layout with the default setting. The dynamical model from scVelo (v0.2.2) was used to estimate RNA velocities and velocity graphs. CellRank (v1.1.0) was performed as described in (https://cellrank.readthedocs.io/en/stable/index.html). A weighted transition matrix with 80% RNA velocity and 20% similarity was used. The Clustering and Filtering of Left and Right Eigenvectors (CFLARE) estimator was used to compute fate probabilities. The terminal states were manually set on the basis of a priori knowledge. Absorption probabilities—how likely each cell is to transition toward each terminal state—were calculated. Pearson’s correlation between absorption probabilities and gene expression levels was computed; genes with high correlation were considered driver genes (data S2). Generalized additive models were used to fit imputed gene expression trends with default settings. The ClusterProfiler package (*79*) was used to examine the enrichment for biological process Gene Ontology Term and Kyoto Encyclopedia of Genes and Genomes pathways of the top 100 driver genes.

### Codependency and mutual exclusivity analysis of scRNA-seq

The proliferative NPCs were digitally isolated from scRNA-seq data, and the coexpressed and mutually exclusive gene pairs were calculated (*29*). The gene pairs containing *Atoh1* or *Ascl1* with high CDI (CDI > 10.0) and EEI (EEI > 3.0) are shown in data S3. The Seurat FeatureScatter function was used to create a scatterplot of two features across the NPCs.

### snATAC-seq and data analysis

Single nucleus isolation for ATAC-seq library generation was performed following the instructions of 10X Genomics. BCL files generated from sequencing were used as inputs to the 10X Genomics Cell Ranger ATAC pipeline (version 1.2.0). FASTQ files were generated and aligned to the mm10 reference genome using BWA. The resultant fragment files were loaded into the ArchR pipeline (v1.0.0) (*80*). A cell-by-bin matrix was generated for each sample by segmenting the genome into 500-bp windows and scoring each cell for reads in each window. Cells were filtered on the basis of log10(UMI) between 2.8 and 5.5, and the fraction of reads in promoters between 12 and 45%. Bins were then filtered by removing those overlapping with the ENCODE blacklist, those mapped to “random” and Y chromosomes, and the top 5% overlapping with invariant features. A cell-by-cell similarity matrix was generated by calculating the latent semantic index (LSI) of the binarized bin matrix. Principal components analysis was then performed on LSI values, and cell clusters were identified with Leiden clustering.

### Defining snATAC-seq cell cluster identity

Gene activity scores were calculated using the ArchR algorithm, which is primarily based on the local accessibility of the promoter and gene body and also takes into account the distal elements (up to 5 kb) and the gene size (*80*). The gene activity scores were normalized by read depth across all genes to a constant of 10,000. The ArchR getMarkerFeatures function was used to select marker genes on the basis of gene activity scores with a cutoff at FDR ≤ 0.01 and log_2_FC ≥ 1.25. To match snATAC-seq and scRNA-seq data, we integrated the derived gene activity scores with gene expression levels using canonical correction analysis to match cells from ATAC to their nearest neighbors in RNA (*77*). Using a modified FindTransferAnchors function of Seurat, ArchR aligned snATAC-seq and scRNA-seq data of mouse cerebella at E12.5, E13.5, and E14.5. As a result, each cell in snATAC-seq was assigned a gene expression signature that was used for downstream analyses.

### ATAC peak calling

Fragments from cells were grouped by cluster, and 501-bp fixed-width peaks were called on all cluster fragments using MACS2 (https://github.com/taoliu/MACS) with the parameters “--nomodel --shift -37 --ext 73 --qval 1e-2 -B -- SPMR --call-summits.” Peaks from each cluster were then combined to form a master peak set, and a cell-by-peak matrix was constructed. This matrix was binarized for all downstream applications.

### Comparison of the global structure of UMAP embedding for RNA and ATAC cells

To avoid confounding batch effects, we focused on the E12.5 data, which reproduced all trajectories of the combined dataset (E12.5 to E14.5). As described previously, we aligned snATAC-seq and scRNA-seq data using the ArchR FindTransferAnchors function. The snATAC-seq object was converted to a Seurat object using custom R scripts. Latent time values from RNA cells were transferred to their nearest ATAC cell neighbors. The Seurat FeaturePlot function was used to create plots for fig. S8 (A and B).

### Determination of differentially accessible peaks and cluster-specific peaks

The ArchR getMarkerFeatures function was used to identify differentially accessible peaks for each cluster with a cutoff at FDR ≤ 0.01 and log_2_FC ≥ 1.0.

### TF motif enrichment analysis

Using motif information from CIS-BP (database 2.0), we used the ArchR addMotifAnnotations function to convert the peak-by-cell matrix into a motif-by-cell binary matrix based on the motif present in the peak. The peakAnnoEnrichment function was used to calculate enrichment for known TF motifs in cluster-specific peaks. To evaluate motif enrichments at the single-cell level based on chromVAR (*35*), the ArchR addDeviationsMatrix function was used to compute per-cell motif deviations.

### Identification and validation of CREs of embryonic mouse cerebellum

The ArchR addPeak2GeneLinks function was used to identify peak-to-gene links with the default settings except for changing the maximal distance between the peak and gene to 500 kb. After identifying 71,162 peak-to-gene links from the merged snATAC-seq dataset, we iterated the analysis and identified additional peak-to-gene links that were specific to each stage (18,158/all; 42,464/E12.5 only; 16,794/E13.5 only; 49,192/E14.5 only), resulting in a total of 197,299 unique peak-to-gene pairs.

The evolutionary conservation scores, phastCons60way and phyloP60way, were retrieved from the UCSC Genome Browser data portal. The average conservation scores in 2 kb centering CRE midpoint were computed using the aggregate function of bwtool (version 1.0) (*81*). Random genomic regions were created by shuffling the original CREs. The proximal enhancer–like (72,794) and distal enhancer–like (209,040) of all mouse CREs (*38*) were downloaded from the SCREEN database (https://screen.encodeproject.org). Permutation tests of overlapping between CREs and other genomic features were performed using the regioneR package.

### Generation and simulation of GRNs with CellOracle

Reconstruction and simulation of GRNs were performed with CellOracle (v0.6.2) following the authors’ instructions (https://morris-lab.github.io/CellOracle.documentation/). On the basis of the inferred CRE-target list, we extracted all potential connections between TFs and targets using the GimmeMotifs motif database with a false-positive rate threshold of 0.02. Top GRN genes were selected and ranked using a combined score of degree centrality, betweenness centrality, and eigenvector centrality. These network scores were normalized between 0 and 1—divided all values by the maximum of the corresponding scores. The average of the scaled scores was used to rank genes in the GRN of each cell type. Cell state transitions resulting from the perturbation of specific TFs were simulated through 5× iterations. For the simulation of the knockout of *Atoh1*, *Ptf1a*, or *Hes1*, we set the expression of the corresponding gene at 0. For the simulation of the GOF of *Hes1*, we set the *Hes1* expression value at 3.0, which was a twofold increase of the detected *Hes1* expression.

### Disease enrichment analysis

The DWM gene list was downloaded from ToppGene Suite. The human genes were converted to the mouse orthologs. The Seurat AverageExpression function was used to output average expression matrix of different cerebellar cell types. The pheatmap R package was used to create heatmaps of the cell type expression of DWM genes. Enrichment testing was performed with Fisher’s exact tests using custom R scripts. The Seurat AddModuleScore function was used to summarize six DWM genes (*Fgf17*, *Flna*, *Lamc1*, *Nphp1*, *Zic4*, and *Zic5*), which are highly expressed in the PTZ, and the aggregated expression—average relative expression as DWM feature—was plotted on UMAP.

## Notes

View/request a protocol for this paper from *Bio-protocol*.
