# Supplementary material for: Integrated single-cell transcriptomic and epigenetic study of cell state transition and lineage commitment in embryonic mouse cerebellum
Source: Sci Adv. 2022 Apr 1;8(13):eabl9156. doi: 10.1126/sciadv.abl9156 (PMC10938588; doi:10.1126/sciadv.abl9156)
Supplement: Supplementary file 1 — Figs. S1 to S13 Table S1 [file sciadv.abl9156_sm.pdf]

Science Advances
AAAS

Supplementary Materials for

Integrated single-cell transcriptomic and epigenetic study of cell state transition and lineage commitment in embryonic mouse cerebellum

Nagham Khouri-Farah, Qiuxia Guo, Kerry Morgan, Jihye Shin, James Y. H. Li*

*Corresponding author. Email: jali@uchc.edu

Published 1 April 2022, *Sci. Adv.* **8**, eabl9156 (2022)
DOI: 10.1126/sciadv.abl9156

The PDF file includes:

Figs. S1 to S13
Table S1
Legends for data S1 to S8

Other Supplementary Material for this manuscript includes the following:

Data S1 to S8

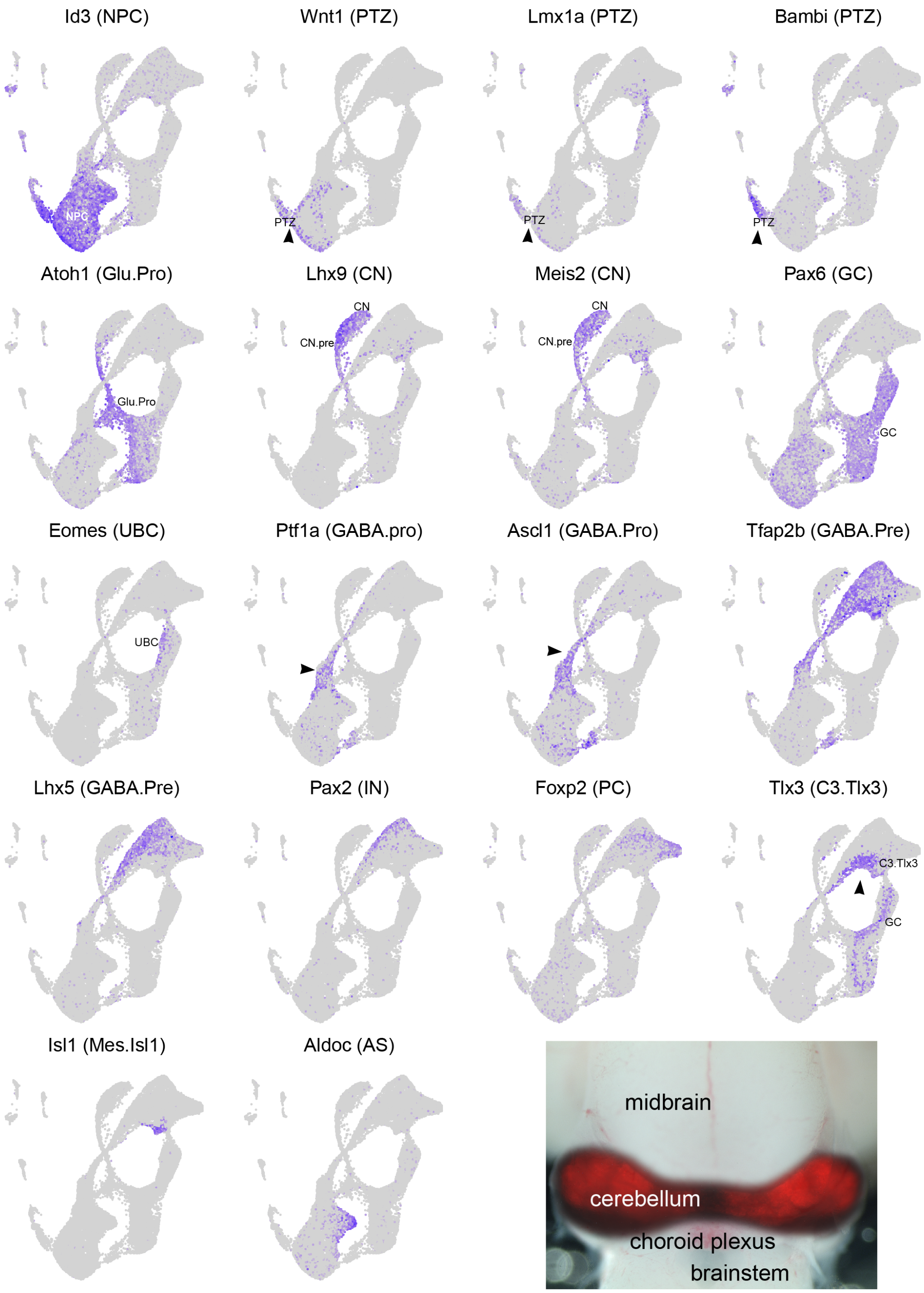

Supplementary Figure S1. **Examination of cell-type specific markers in E10.5-E17.5 cerebellar scRNA-seq**. Expression of cerebellar cell-type-specific markers. Abbreviations: BG/AS, Bergmann glia and astrocytes; Eryth.early and Eryth.late, early and late erythrocytes; GABA.Pro, GABAergic neuron progenitors; GABA.Pre, GABAergic neuron precursors; Glu.Pro, glutamatergic neuron progenitors; Isth, isthmus; GC, granule cell; GCP, granule cell progenitor; NPCa, NPCs of the anterior part of the cerebellar VZ; NPC.M and NPC.S, NPCs in the M and S phase; PTZ, posterior transitory zone. Dorsal view of the mouse embryonic cerebellum (in red) is shown in the lower right corner.

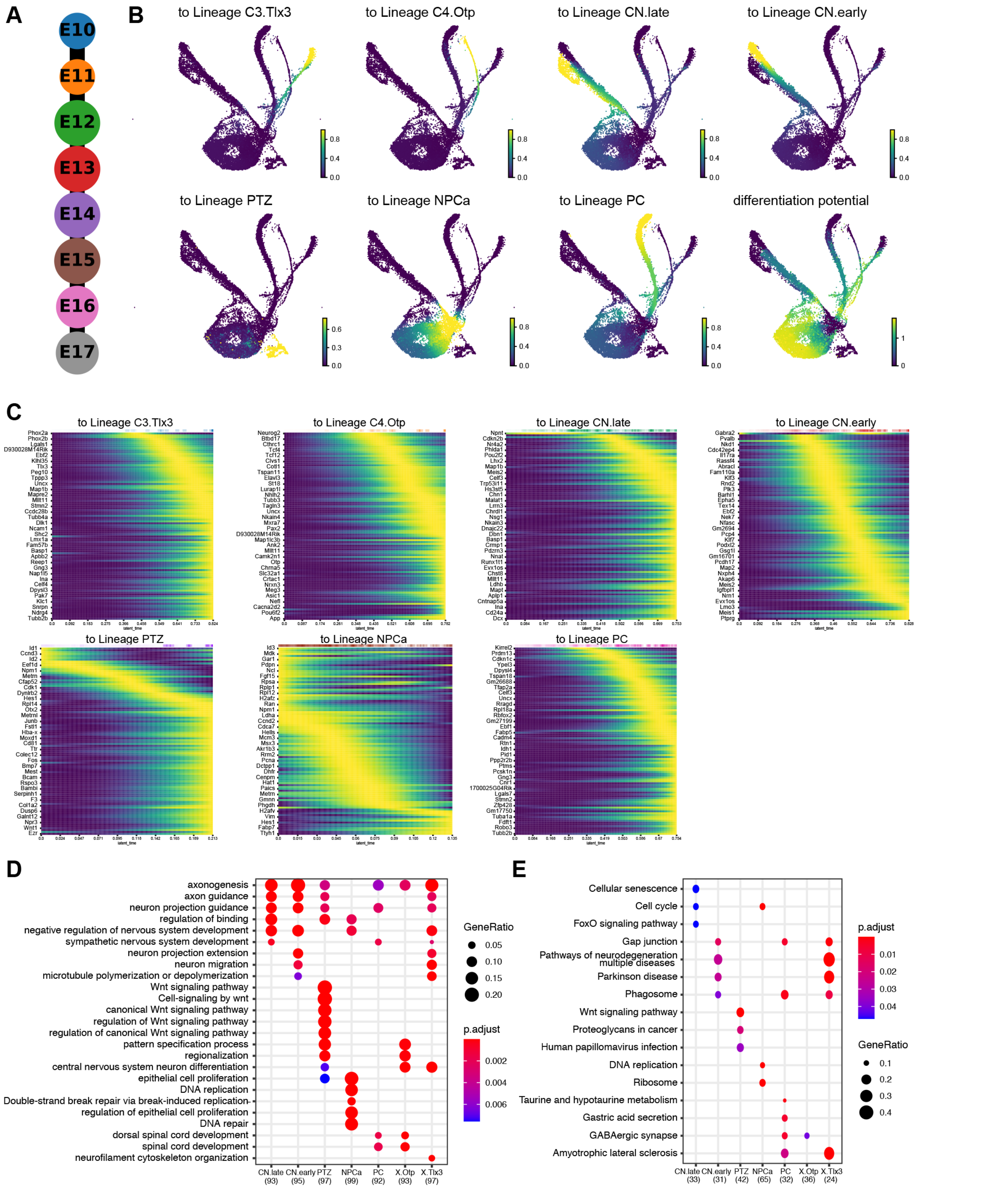

Supplementary Figure S2. Inference of cerebellar development with scRNA-seq. (A) Unbiased hierarchical ordering of cerebellar cells from different embryonic stages by PAGA. (B) Cell-fate probabilities inferred by CellRank in different lineages. (C) Heatmaps showing the

expression dynamics of top 100 driver genes in different lineages. Dot plots showing enrichment for GO terms (D) and KEGG pathways (E) of top 100 driver genes.

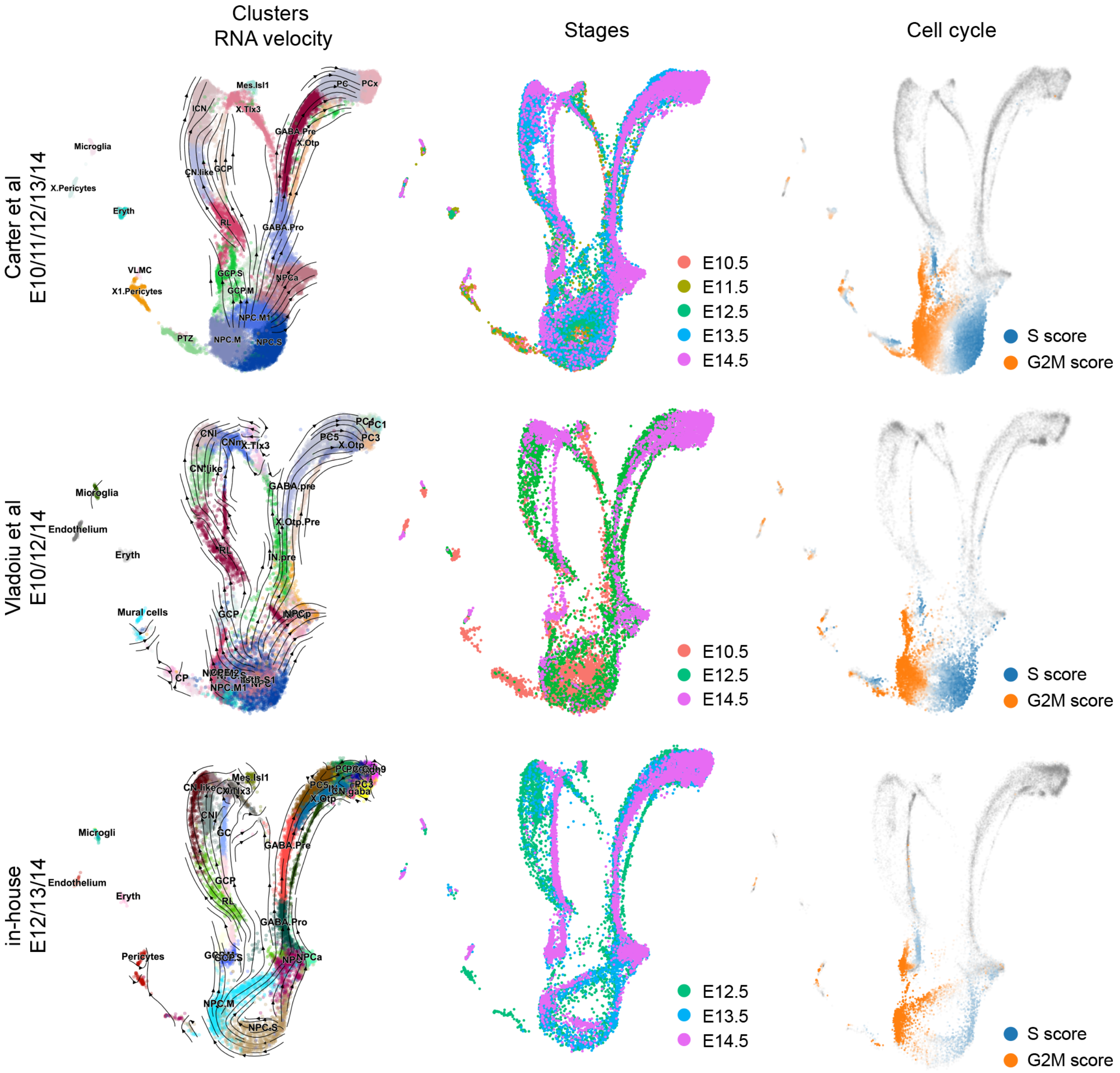

Supplementary Figure S3. **Assessment of the robustness of scRNA-seq analyses.** UMAP showing cell clusters, RNA velocity streams, stages, and cell cycles of three different scRNA-seq datasets.

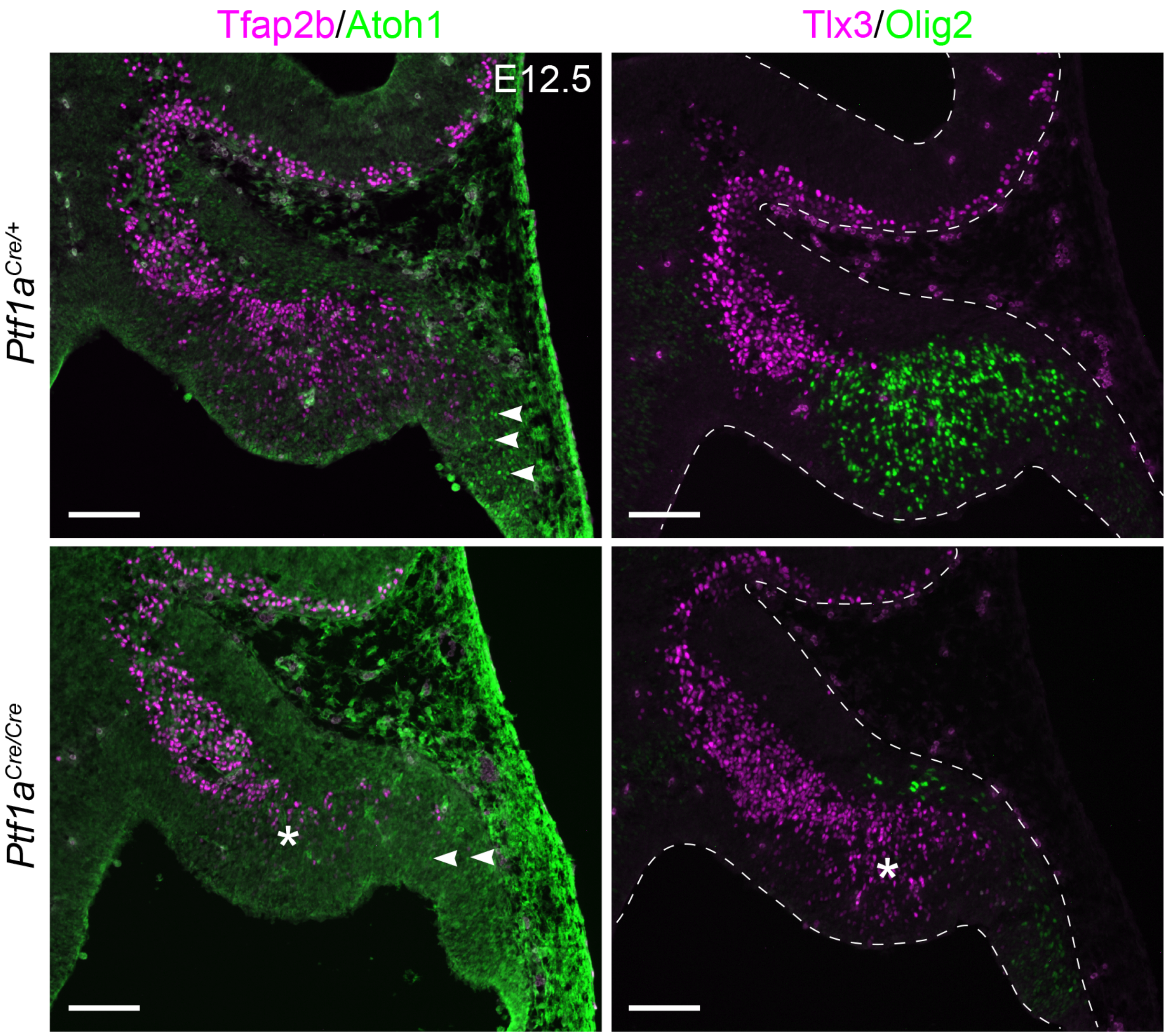

Supplementary Figure S4. **Conversion of the C2 fate to C3 in the cerebellar anlage without *Ptf1a***. Immunofluorescence on sagittal sections of E12.5 cerebella of the indicated genotype. Arrowheads indicate nascent Atoh1-expressing cells; the asterisk denotes the reduction of Tfap2b and the absence of Olig2 in the C2 area. Scale bars are 100 μm.

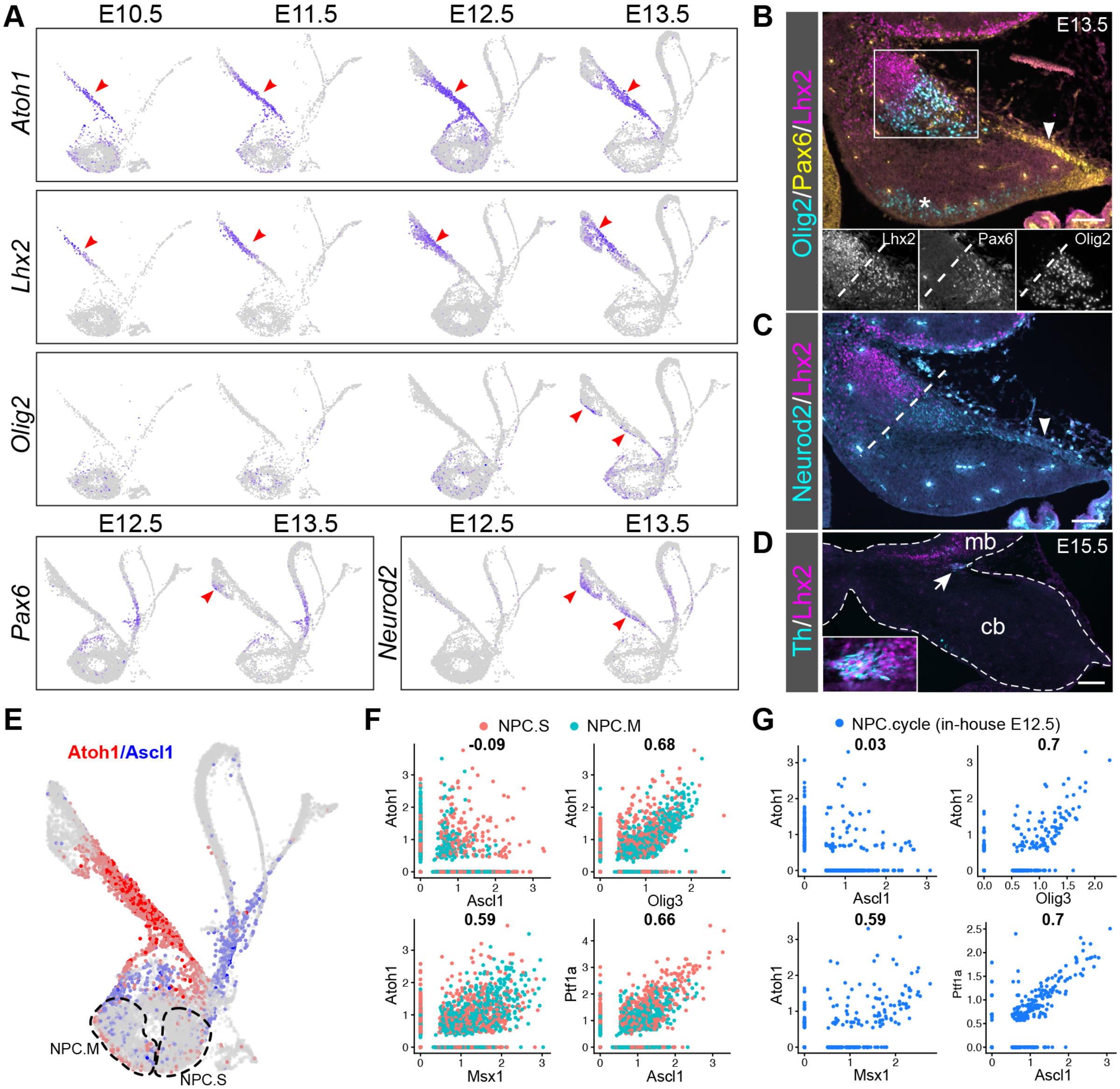

Supplementary Figure S5. **Analysis of the *Atoh1* lineage.** (A) Expression of CN.early and CN.late markers. (B and C) Immunofluorescence on sagittal sections of E13.5 cerebella. The dashed lines demarcate the anterior and posterior parts of the nuclear transitory zone. The boxed area is shown in individual channels below B. (D) Immunofluorescence on coronal sections of the E15.5 cerebellum. The arrow indicates isthmic nucleus that is labeled by both Lhx2 and Th (enlarged in the inset). (E) UMAP showing expression of *Atoh1* and *Ascl1* in scRNA-seq of E10.5-E13.5 cerebella. Note the sporadic *Aoth1*$^+$ and *Ascl1*$^+$ cells among cycling NPCs in the M and S phase (NPC.M and NPC.S). (F and G) Scatter plots of expression of *Atoh1*, *Ascl1*, and other genes across proliferative NPCs from Carter's E10.5-E13.5 (F) and in-house E12.5 (G) datasets. Pearson correlations between the two features is displayed above each plot. Scale bars are 100 µm (B and C) and 200 µm (D).

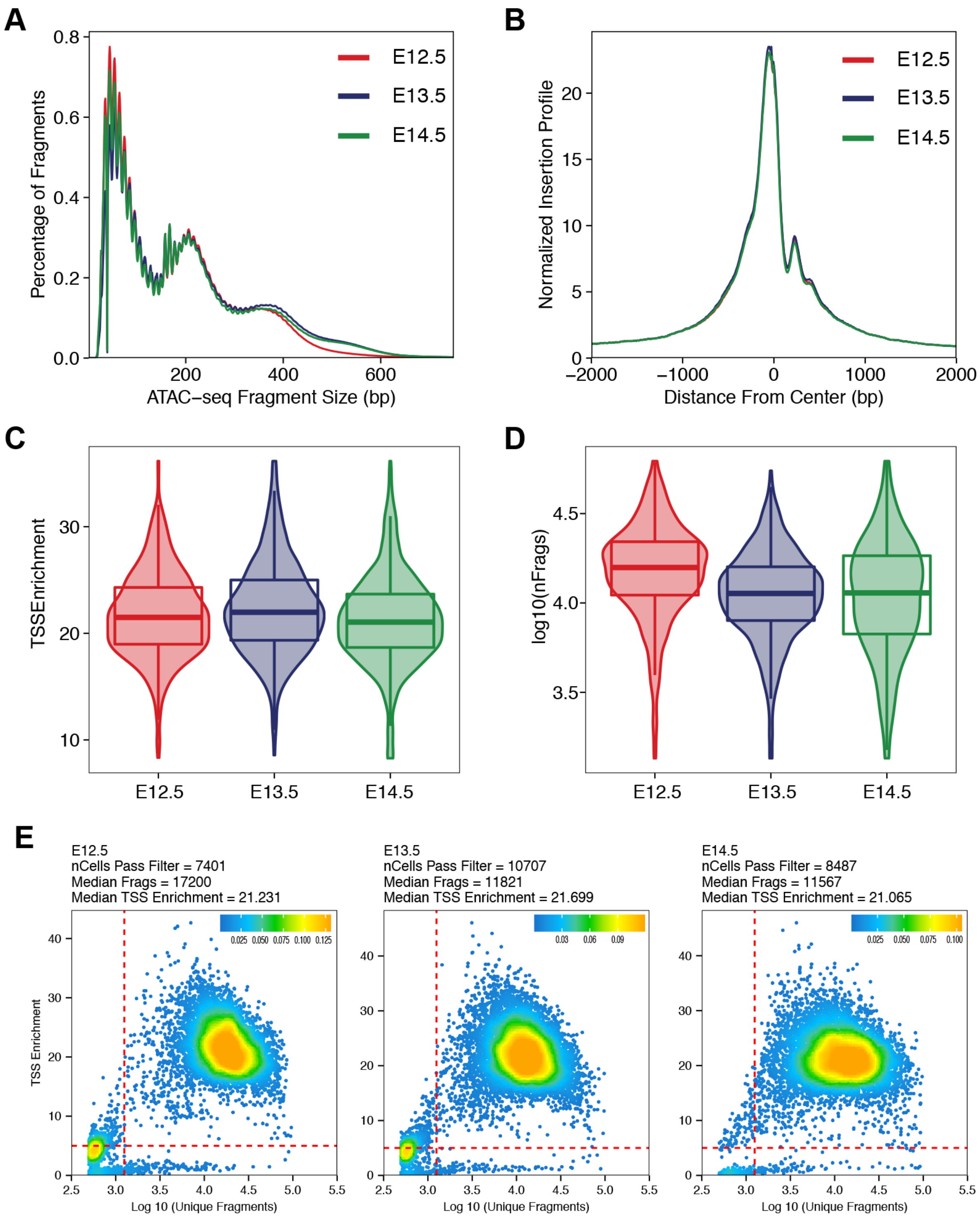

Supplementary Figure S6. **Data quality of the snATAC-seq libraries**. Line plots showing the fragment size distribution (A) and fragment distribution around the transcription start site (TSS; B). Boxplots comparing the TSS enrichment (C) and total fragments (D) of different experiments. (E) Scatter plots showing filtering of snATAC-seq cells based on TSS enrichments and total fragments. The red dashed lines indicate the filtering threshold.

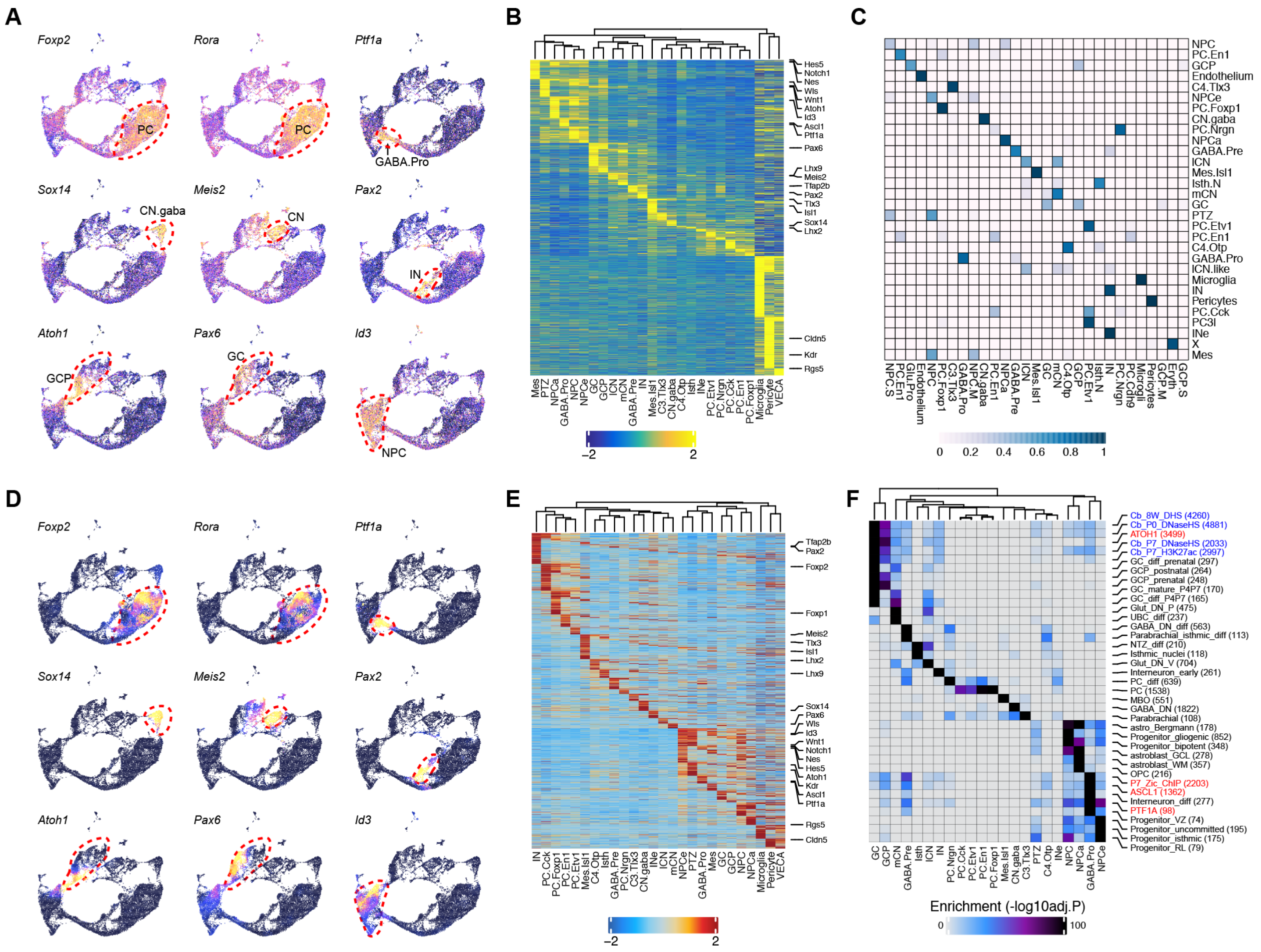

Supplementary Figure S7. **Linkage of ATAC cells with RNA cells**. UMAP (A) and heatmap (B) showing marker expression based on gene activity scores. Each cerebellar cell type is circled by red dash lines. (C) Heatmap showing the alignment between scATAC-seq and scRNA-seq clusters. UMAP (D) and heatmap (E) showing linked gene expression calculated based on integrated snATAC-seq and scRNA-seq. Note the similar pattern between A and D, whereas the latter displays more dynamic differences between cell clusters. (F) Heatmap showing enrichments of cell-specific peaks with various genomic features published previously. Published ChIP-seq data are in red; bulk DNAse I hypersensitive site-seq from ENCODE in blue; cell-specific peaks from a previous snATAC-seq in black.

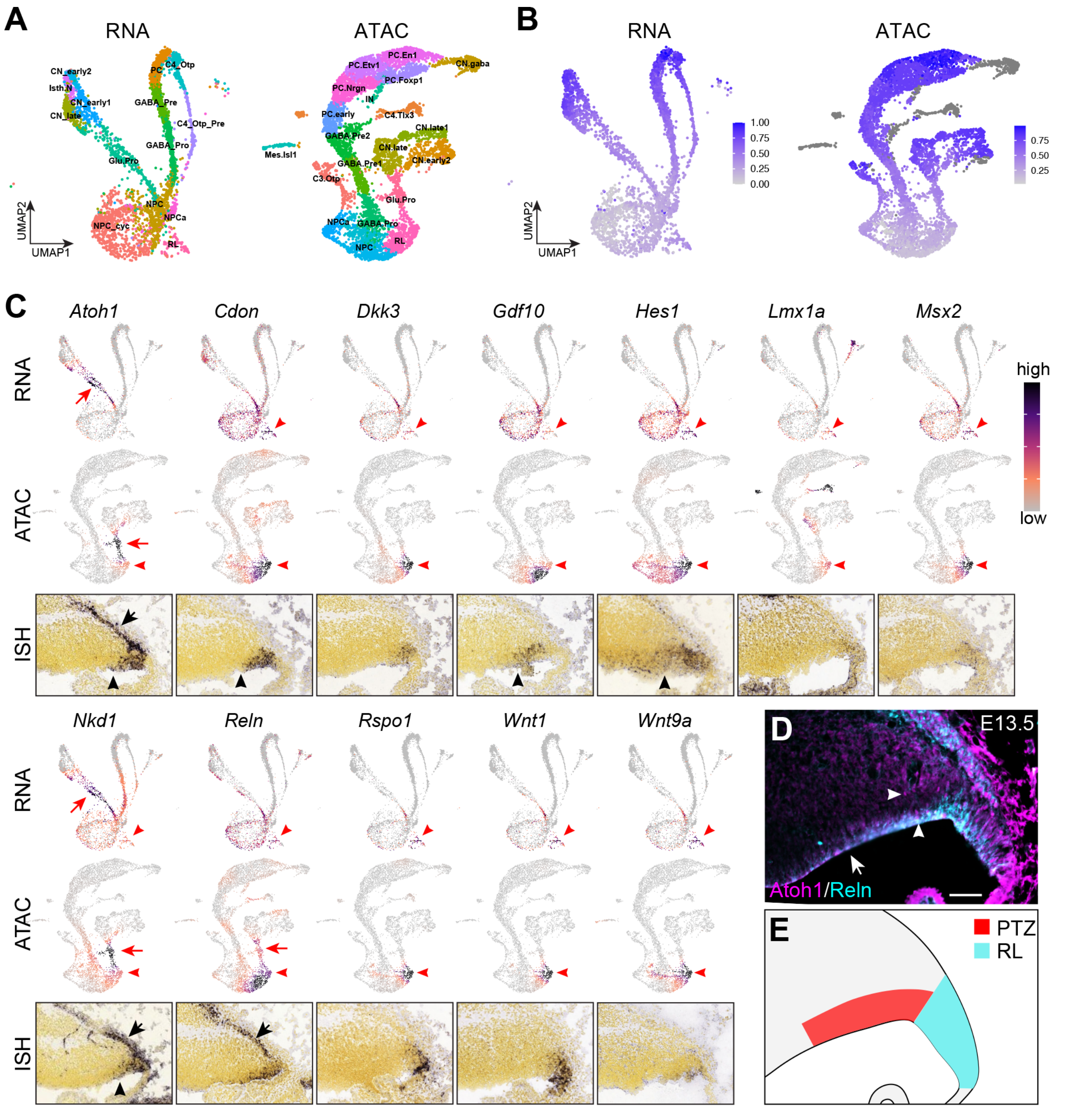

Supplementary Figure S8. Characterization of the molecular feature of PTZ cells with scRNA-seq and snATAC-seq. (A and B) UMAP embeddings of scRNA and snATAC cells from E12.5 mouse cerebella exhibit similar global structure (A) and developmental continuum (B) as measured by latent time inferred by scVelo. **(C)** Inspection of feature genes of the PTZ in scRNA, snATAC cells, and *in situ* hybridization on sagittal sections of E13.5 cerebella (from Allen Developing Mouse Brain Atlas). Arrowheads indicate the PTZ; arrows show glutamatergic progenitors. **(D)** Immunofluorescence on sagittal section of the E13.5 cerebellum. The arrowhead indicates the anterior limit of the PTZ; arrowheads denote nascent Atoh1^{+} cells. (E) Illustration comparing the PTZ and RL domains. The scale bar in D is 50 µm.

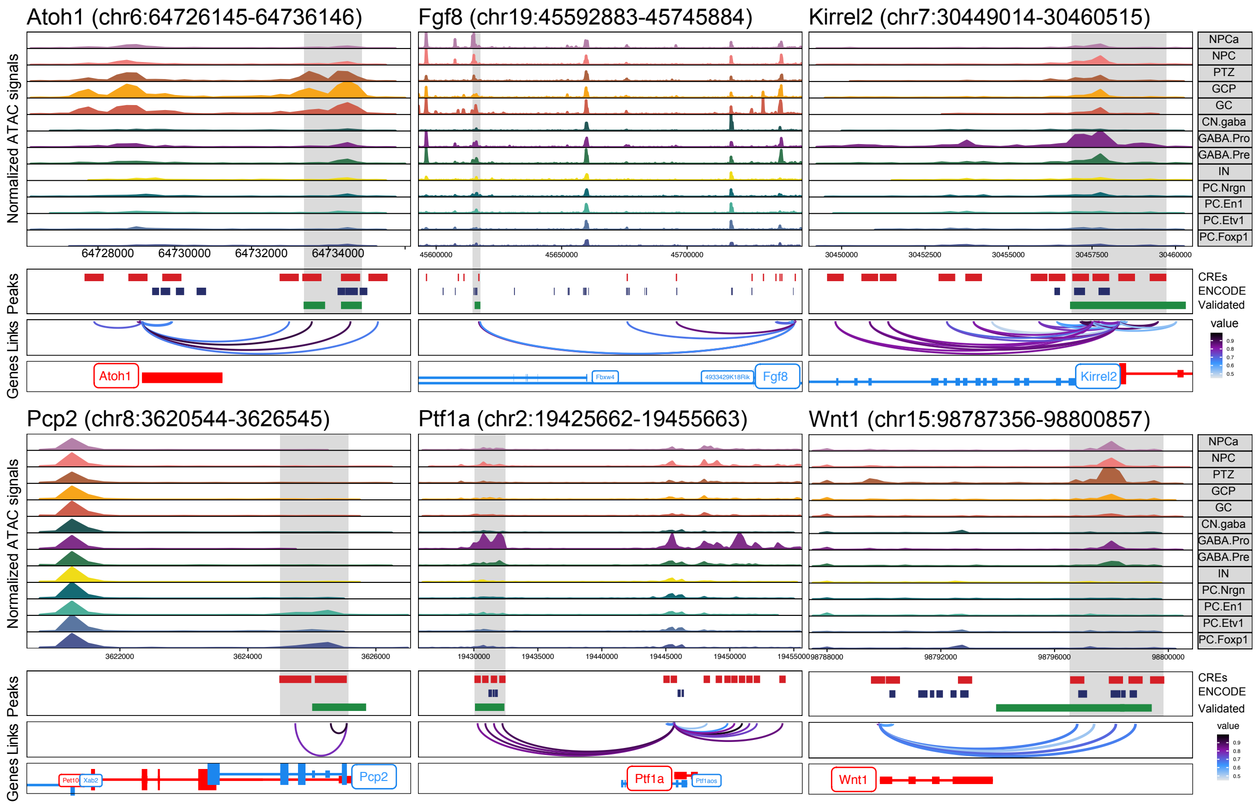

Supplementary Figure S9. Overlapping between CREs and validated enhancers. Examples of linkages of CREs and targets. The grey areas highlight the CRE that has been validated by transgenic mouse assays.

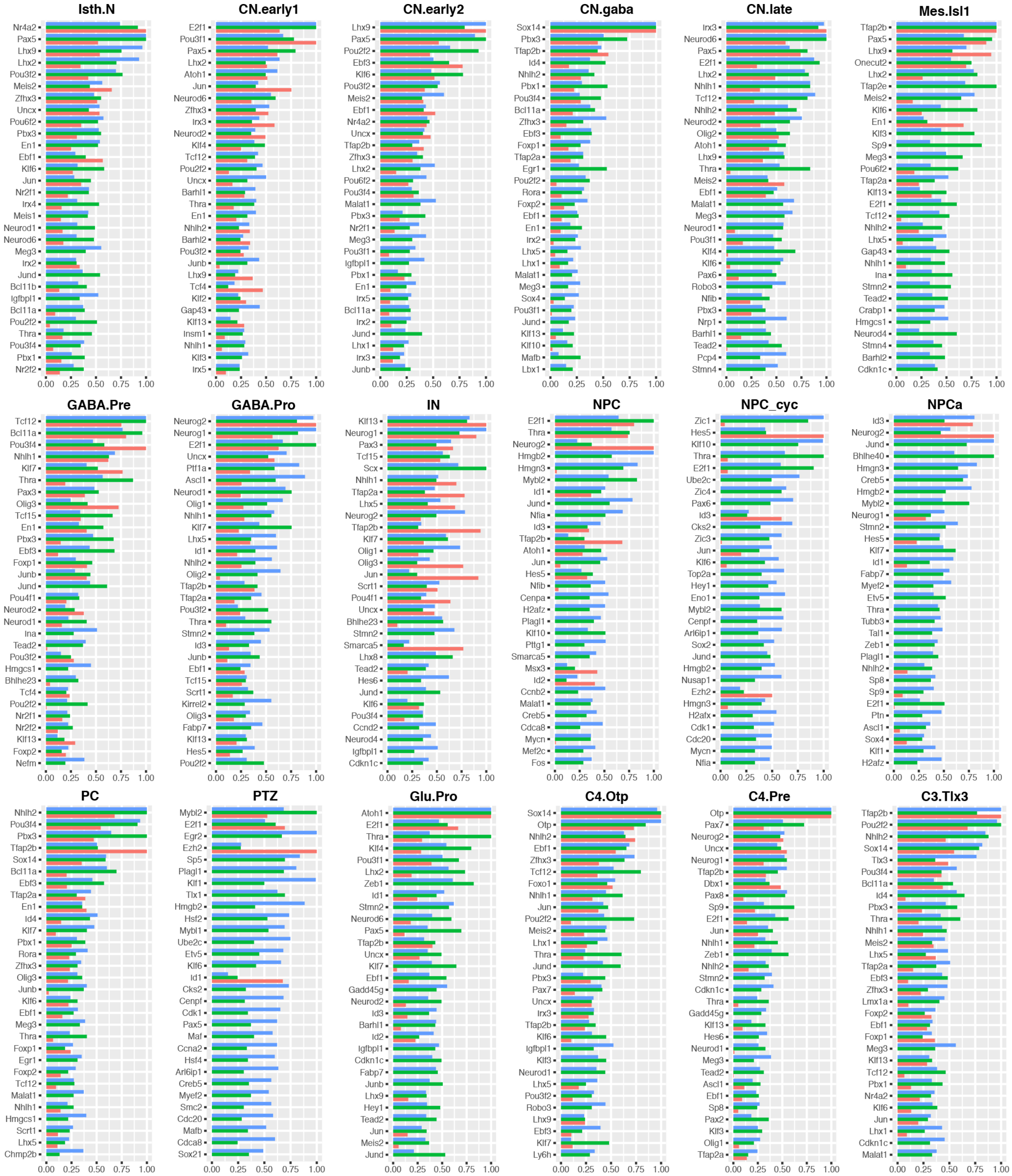

Supplementary Figure S10. **Identification of putative key regulators through network analysis of the GRN specific to each cell type or state**. Top 30 genes based on the aggregate ranking of three key network scores: degree centrality, betweenness centrality, and eigenvector centrality, in the GRN for each cell cluster.

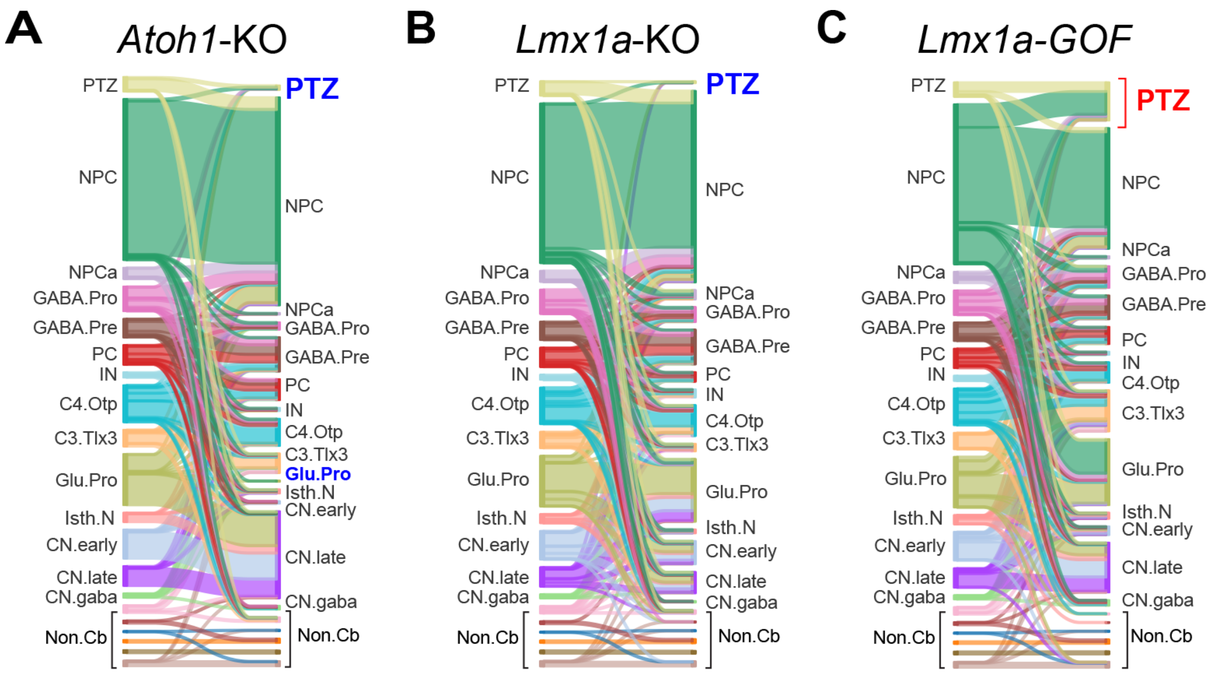

Supplementary Figure S11. **Simulation of genetic perturbations of *Atoh1*, *Ptf1a, and Lmx1a* during cerebellar development**. (A-C) Sankey diagrams showing simulated cell transition between different cell types caused by genetic perturbations indicated on the top. Expanded or reduced cell types are shown in red and blue, respectively.

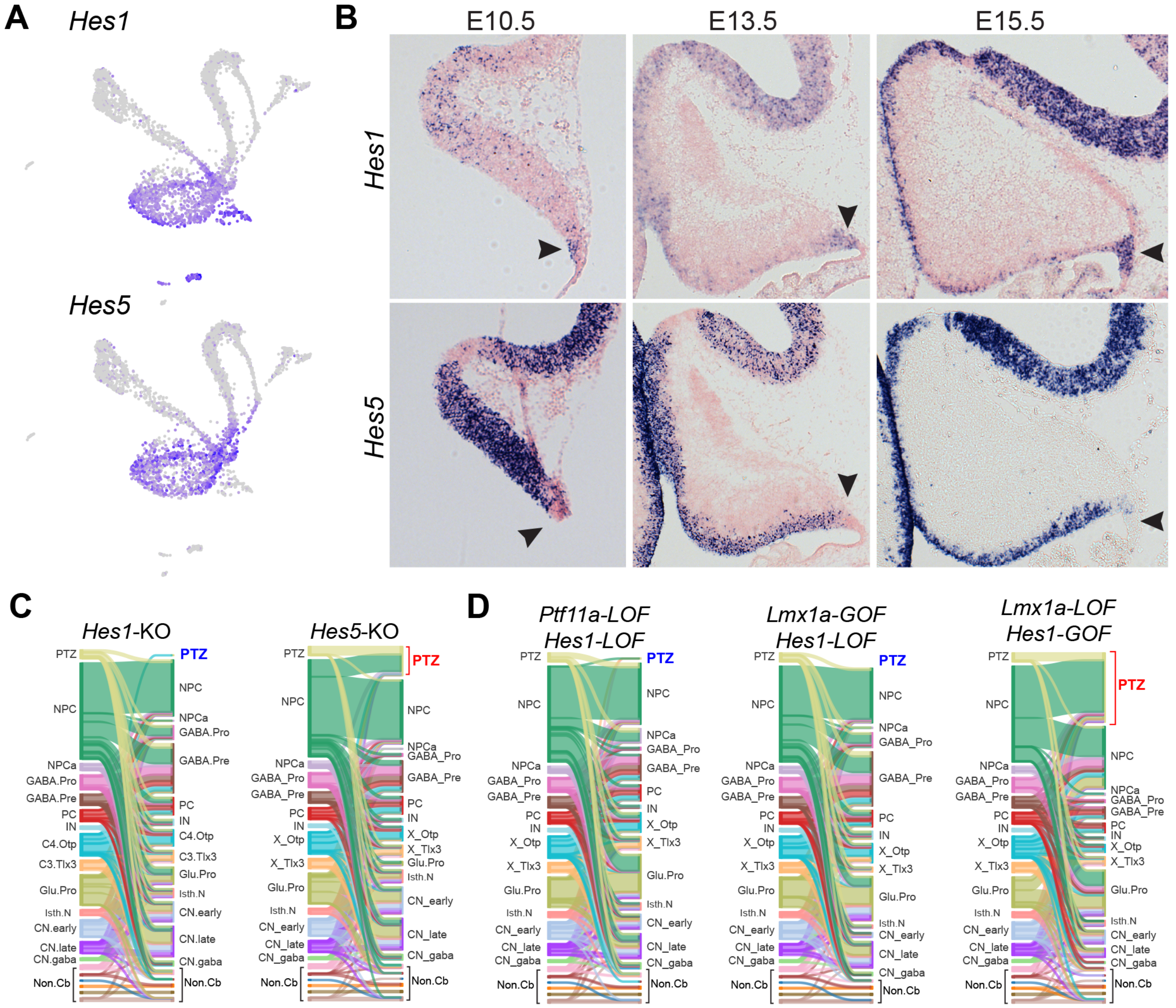

Supplementary Figure S12. **Important role of *Hes1* in PTZ development**. (A and B) Expression of *Hes1*, but not *Hes5*, in the PTZ as shown by scRNA-seq of E12.5 cerebella (A) and *in situ* hybridization (B). (C and D) Sankey diagrams showing simulated cell transition between different cell types caused different mutant genotypes indicated on the top. The expanded or reduced/lost PTZ is shown in red and blue, respectively.

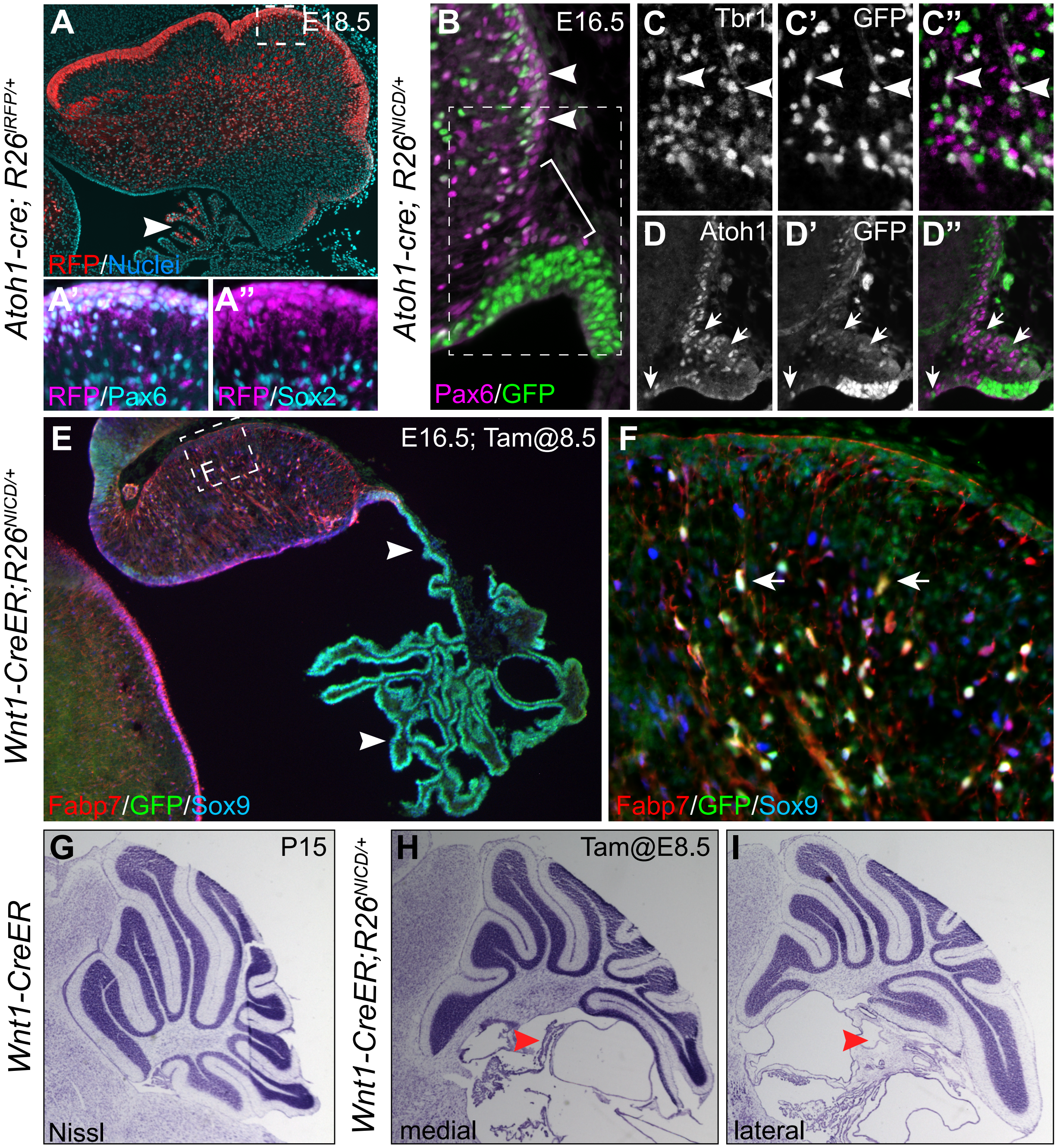

Supplementary Figure S13. **Notch activation alters cell fate specification of PTZ and results in truncation of the posterior cerebellar vermis**. (**A-A"**) Red fluorescence and immunofluorescence showing the distribution of *Atoh1*-lineage descendants in the cerebellum. (**B-D"**) Immunofluorescence on sagittal section of *Atoh1-Cre; $R26^{NICD/+}$* cerebella at E16.5.; The bracket shows the discontinuous EGL next to the RL; arrowheads denote *$NICD^{+}$* cells marked by GFP in the EGL (B) and fastigial nucleus (C-C"); Arrows indicate the lack of *Atoh1* immunoreactivity in *$NICD^{+}$* cells at the PTZ. (**E and F**) Immunofluorescence on a sagittal section of E16.5 *Wnt1-CreER;$R26^{NICD/+}$* embryos that were given tamoxifen at E8.5. The arrowhead indicates the enlarged choroid plexus; Arrows denote Bergmann glia with NICD

expression. (**G-I**) Nissl histology of sagittal sections of the cerebellar vermis of indicated genotypes at P15. The arrowhead shows the enlarged choroid plexus. Note that the posterior truncation is more prominent in the medial section (H) than the lateral (I) part of the cerebellar vermis.

Supplementary Table S1

Stage	E10.5	E11.5	E12.5	E13.5	E14.5	E15.5	E16.5	E17.5	Total
Carter's	2,716	2,459	7,033	7,189	7,107	7,233	5,109	5,356	44,202
Vladoiu's	5,593		6,329		5,233				17,155
In-house			11,636	9,178	14,420				35,234
scATAC			6,864	9,564	7,777				24,205

Supplementary Data 1: Lists of molecular features of different cell types and states of embryonic mouse cerebella (E10.5-E17.5/sheet1 and E10.5-E13.5/sheet2).

Supplementary Data 2: List of driver genes identified by CellRank.

Supplementary Data 3: Lists of co-expressed gene pairs (sheet = CDI) and exclusively expressed gene pairs (sheet = EEI).

Supplementary Data 4: Lists of cluster-specific markers based on gene activity scores (sheet = GeneScoreMatrix) and RNA-integrated expression (sheet = GeneIntegratedMatrix) in snATAC-seq.

Supplementary Data 5: List of putative transcription activators and repressors in early cerebellar development.

Supplementary Data 6: List of top 10 percentile genes with the highest number of CREs and results of GO term enrichment analysis.

Supplementary Data 7: List of CellOracle-inferred key regulators of different cell groups.

Supplementary Data 8: List of antibodies used in this study.
